# Consensus Guidelines for Perioperative Care in Neonatal Intestinal Surgery: Enhanced Recovery After Surgery (ERAS^®^) Society Recommendations

**DOI:** 10.1007/s00268-020-05530-1

**Published:** 2020-05-08

**Authors:** Mary E. Brindle, Caraline McDiarmid, Kristin Short, Kathleen Miller, Ali MacRobie, Jennifer Y. K. Lam, Megan Brockel, Mehul V. Raval, Alexandra Howlett, Kyong-Soon Lee, Martin Offringa, Kenneth Wong, David de Beer, Tomas Wester, Erik D. Skarsgard, Paul W. Wales, Annie Fecteau, Beth Haliburton, Susan M. Goobie, Gregg Nelson

**Affiliations:** 1 Department of Surgery, Alberta Children’s Hospital, Cumming School of Medicine, University of Calgary, 28 Oki Drive, Calgary, AB T3B 6A8 Canada; 2 Department of Medicine, McMaster University, 1280 Main Street West, Hamilton, ON L8S 4L8 Canada; 3 Department of Anesthesiology, Children’s Hospital Colorado, 13123 E 16th Avenue, Aurora, CO 80045 USA; 4 Division of Pediatric Surgery, Department of Surgery, Northwestern University Feinberg School of Medicine and Ann & Robert H. Lurie Children’s Hospital of Chicago, 225 E Chicago Avenue, Chicago, IL 60611 USA; 5 Section of Neonatology, Department of Pediatrics, Alberta Children’s Hospital, Cumming School of Medicine, University of Calgary, 28 Oki Drive, Calgary, AB T3B 6A8 Canada; 6 Division of Neonatology, Hospital for Sick Children, University of Toronto, 555 University Avenue, Toronto, ON M5G 1X8 Canada; 7 Department of Surgery, Li Ka Shing Faculty of Medicine, University of Hong Kong, 21 Sassoon Road, William M. W. Mong Block, Hong Kong, China; 8 Department of Pediatric Anesthesia, Great Ormond Street Hospital, Great Ormond Street, London, WC1N 3JH UK; 9 Department of Pediatric Surgery, Karolinska University Hospital, Eugeniavagen 23, 171 76 Solna, Stockholm, Sweden; 10 Department of Pediatric Surgery, British Columbia Children’s Hospital, 4480 Oak Street, Vancouver, BC V6H 3N1 Canada; 11 Division of General and Thoracic Surgery, Hospital for Sick Children, University of Toronto, 555 University Avenue, Toronto, ON M5G 1X8 Canada; 12 Department of Anesthesiology, Critical Care and Pain Medicine, Boston Children’s Hospital, Harvard University, 300 Longwood Avenue, Boston, MA 02115 USA; 13 Department of Oncology, Cumming School of Medicine, University of Calgary, 1403 29 Street NW, Calgary, AB T2N 2T9 Canada; 14 Department of Surgery and Community Health Sciences, Cumming School of Medicine, University of Calgary, 28 Oki Drive, Calgary, AB T3B 6A8 Canada

## Abstract

**Background:**

Enhanced Recovery After Surgery (ERAS^®^) Society guidelines integrate evidence-based practices into multimodal care pathways that have improved outcomes in multiple adult surgical specialties. There are currently no pediatric ERAS^®^ Society guidelines. We created an ERAS^®^ guideline designed to enhance quality of care in neonatal intestinal resection surgery.

**Methods:**

A multidisciplinary guideline generation group defined the scope, population, and guideline topics. Systematic reviews were supplemented by targeted searching and expert identification to identify 3514 publications that were screened to develop and support recommendations. Final recommendations were determined through consensus and were assessed for evidence quality and recommendation strength. Parental input was attained throughout the process.

**Results:**

Final recommendations ranged from communication strategies to antibiotic use. Topics with poor-quality and conflicting evidence were eliminated. Several recommendations were combined. The quality of supporting evidence was variable. Seventeen final recommendations are included in the proposed guideline.

**Discussion:**

We have developed a comprehensive, evidence-based ERAS guideline for neonates undergoing intestinal resection surgery. This guideline, and its creation process, provides a foundation for future ERAS guideline development and can ultimately lead to improved perioperative care across a variety of pediatric surgical specialties.

**Electronic supplementary material:**

The online version of this article (10.1007/s00268-020-05530-1) contains supplementary material, which is available to authorized users.

## Introduction

Enhanced Recovery After Surgery (ERAS^®^) guidelines are designed to deliver standardized, evidence-based, collaborative care throughout the surgical journey [1–6]. The ERAS^®^ Society published its first guideline in adult colorectal surgery which was subsequently adapted for use in other surgeries [1]. ERAS^®^ implementation has reduced complications, length of stay (LOS), and costs, while improving patient and staff satisfaction [5–7]. Despite these successes, there are few pediatric ERAS^®^ studies and no ERAS^®^ Society pediatric guidelines. Limited applications of ERAS^®^ in children have demonstrated reduced surgical infections, readmissions, reoperations, LOS, and cost [4, 8–10].

Neonates could greatly benefit from ERAS^®^ as they experience variable perioperative care and suffer high rates of complications [11, 12]. Neonatal ERAS^®^ guidelines must consider the unique aspects of neonatal physiology as well as a unique perioperative team [13–15].

Our international team collaboratively developed the first ERAS^®^ guideline for surgical neonates using a rigorous, evidence-based, consensus-driven process integrating parents and clinicians.

## Methods

The details of our approach have been published and are summarized below [16].

### Multidisciplinary team

A guideline development committee (GDC) was assembled including surgeons, anesthesiologists, and neonatologists as well as subject matter experts. Parent representatives were consulted at multiple points during guideline development.

### Scope determination

A modified Delphi method was used to reach consensus regarding the target population and topics. The target population was determined to be term neonates (≥37 weeks gestational age) without major comorbidities undergoing intestinal resection surgery within the first 4 weeks of life. Complex surgical conditions were excluded including necrotizing enterocolitis (NEC), abdominal wall defects, and short bowel syndrome. Fourteen topics were identified for areas of recommendation development (Online resource 1) [16].

### Literature search

GDC members were assigned topics based on expertise. For each topic, a systematic search strategy was performed in conjunction with a research librarian (Online resource 2). Screening followed standard Preferred Reporting Items for Systematic Review and Meta-Analysis (PRISMA) methods [17]. GDC members supplemented these searches with further targeted literature searches [16].

### Study selection and data synthesis

Systematic reviews, randomized and non-randomized controlled trials, observational cohorts and case series were included. Case studies and expert opinion were excluded. Articles meeting eligibility criteria were reviewed in full text. One or more recommendations were drafted for each topic. Evidence was summarized, and the Grading of Recommendations, Assessment, Development and Evaluation (GRADE) approach was used to assess the quality of evidence based on risk of bias, imprecision, inconsistency, indirectness, and publication bias [18].

### Recommendation grading

A two-round modified Delphi was used to review and select recommendations [19]. In the first round, the GDC provided feedback on recommendations and evidence, and rated necessity for inclusion. At the second round, recommendation inclusion in the guideline was determined through consensus. Included items were assessed for aggregate evidence quality and the strength of the recommendation according to the GRADE approach [18] (see Table 1a and b). The strength of the recommendation (“strong” or “weak”) was based on evidence quality, as well as potential desirable and undesirable consequences of the recommendation [18]. Recommendations were reviewed by experts and future guideline users to ensure feasibility.

**Table 1:** GRADE system for rating (a) quality of evidence [18] and (b) strength of recommendations [18]

(a) Quality of evidence	Definition
High quality	Further research is very unlikely to change confidence in the estimate of effect
Moderate quality	Further research is likely to have an important impact on confidence in the estimate of effect and may change the estimate
Low quality	Further research is very likely to have an important impact on confidence in the estimate of effect and is likely to change the estimate
Very low quality	Any estimate of effect is very uncertain

(b) Recommendation strength	Definition
Strong	When the desirable effects of an intervention clearly outweigh the undesirable effects, or clearly do not
Weak (“conditional” or “discretionary”)	When the trade-offs are less certain—either because of low-quality evidence or because evidence suggests that desirable and undesirable effects are closely balanced

## Results

Of 3514 total publications reviewed, 2909 were identified via initial systematic searches and 605 through additional searches, citation searches, and expert identification. Screening and data extraction was performed within each topic (Online resource 3). GDC members reviewed the evidence and submitted 36 preliminary recommendations.

Based on consensus, recommendations were eliminated due to very poor-quality or conflicting evidence. Other recommendations were rephrased or combined.

**Fig. 1:**
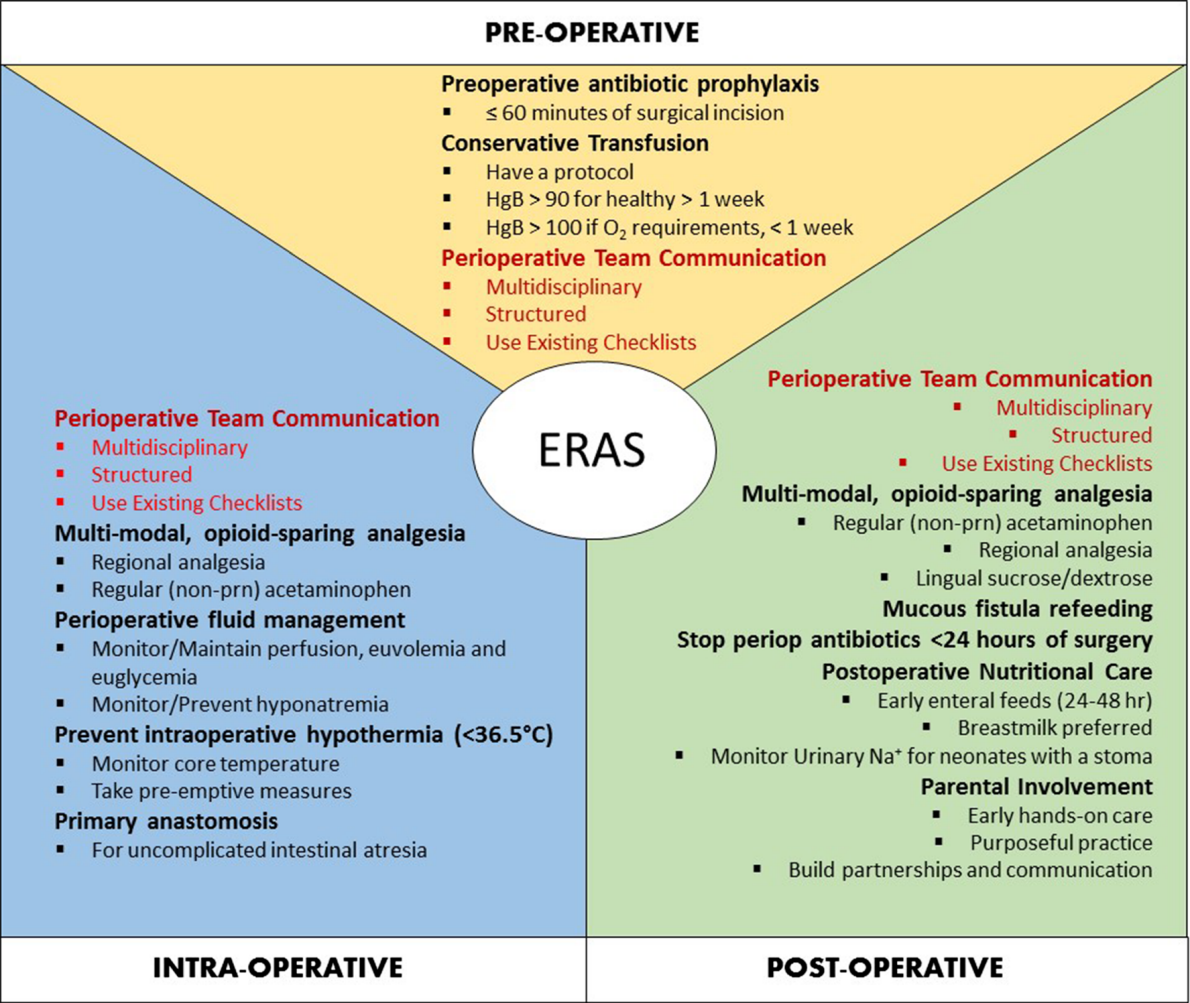
Elements of the ERAS^®^ approach for neonatal intestinal resection surgery. Refer to Table 2 for greater detail on each recommendation

The final ERAS^®^ guideline has 17 recommendations (Table 2) (Fig. 1). Overall, 116 articles were used to support the recommendations (Online Resource 4). The quality of evidence of these papers was relatively low. 68.1% (*n* = 81) of the papers had a rating of very low (23%; *n* = 27) or low (45%; *n* = 54), 22% (*n* = 26) had a rating of moderate, and 10% (*n* = 12) had a rating of high.

**Table 2:** Guidelines for enhanced perioperative care in neonatal intestinal surgery: Enhanced Recovery After Surgery (ERAS®) Society recommendations

Item	Recommendation	Quality	Strength
Surgical practices	Perform primary anastomosis as the first choice in patients with uncomplicated intestinal atresia	Very low	Weak
Antimicrobial prophylaxis	Administer appropriate preoperative antibiotic prophylaxis within 60 min prior to skin incision	Low	Weak
	Discontinue postoperative antibiotics within 24 h of surgery, unless ongoing treatment is required	Low	Weak
Preventing intraoperative hypothermia	Continuously monitor intraoperative core temperature and take pre-emptive measures to prevent hypothermia (<36.5 °C) and maintain normothermia	Low	Strong
Perioperative fluid management	Use perioperative fluid management to maintain tissue perfusion and prevent hypovolemia, fluid overload, hyponatremia, and hyperglycemia	Moderate	Weak
Perioperative analgesia	Unless contraindicated, administer acetaminophen regularly during the early postoperative period (not on an “as needed” basis) to minimize opioid use	High	Strong
	Use an opioid-limiting strategy is recommended in the postoperative period. Manage breakthrough pain with the lowest effective dose of opioid with continuous monitoring	Moderate	Strong
	Use regional anesthesia and acetaminophen perioperatively in combination with general anesthesia. Multimodal strategies including regional techniques should be continued postoperatively	High	Strong
	Provide lingual sucrose/dextrose to reduce pain during naso/orogastric tube placement and other minor painful procedures	High	Strong
Optimal Hemoglobin	Restrict transfusions to maintaining HgB ≥ 90 (9 g/dL for a term neonate with no oxygen requirement. Term neonates within the first week of life, intubated or with an oxygen requirement should be transfused to maintain a HgB ≥ 110 (11 g/dL)	Low	Weak
	Use written transfusion guidelines and take into account not only a target hemoglobin threshold, but also the clinical status of the neonate and local practices	Low	Weak
Perioperative Communication	Implement perioperative multidisciplinary team communication with a structured process and protocol (“pre- and postoperative huddle”) utilizing established checklists	Moderate	Strong
Parental involvement	Facilitate hands on care and purposeful practice by parents that is individualized to meet the unique needs of parents early during the admission. Sustain these to build the knowledge and skills of parents to take on a leading role as caregivers and facilitate their readiness for discharge	High	Strong
Postoperative nutritional care	Start early enteral feeds within 24-48 h after surgery when possible. Do not wait for formal return of bowel function	High	Weak
	Use breast milk as the first choice for nutrition	High	Strong
	Monitor urinary sodium in all neonates with a stoma. Target urinary sodium should be greater than 30 mmol/L and exceed the level of urinary potassium	Low	Weak
Mucous fistula refeeding	Use mucous fistula refeeding in neonates with enterostomy to improve growth	Moderate	Weak

### Evidence base and recommendations (online resource 5)

#### Surgical practices

In the setting of intestinal atresia, pediatric surgeons must decide between stoma creation or primary anastomosis. Very low quality evidence comparing primary and secondary anastomosis in neonates with intestinal atresia demonstrates that primary anastomosis is associated with a reduction in LOS, decreased readmissions, and decreased need for reoperations [20–22]. Given the likelihood of selection bias in these studies, the recommendation has been limited to neonates with uncomplicated atresia.

**Table:** 

*Recommendation:*	**Perform primary anastomosis as the first choice in patients with uncomplicated intestinal atresia**
*Evidence Quality:*	Very low
*Recommendation Strength:*	Weak

### Antimicrobial prophylaxis

#### Antibiotics within 60 min

Surgical Site Infection (SSI) rates are high in neonatal intestinal surgery, and the consequences of SSIs are severe [11]. The quality of evidence for antibiotic prophylaxis in neonatal surgery is low. Given neonatal pharmacodynamics, SSI rates, and the immunocompromised state of neonates, recommendations were deemed reasonable to extrapolate from the adult literature. High-quality evidence from adult studies demonstrates decreased SSI rates in intestinal surgery patients provided with well-timed preoperative antibiotics [23, 24]. This can be extrapolated to neonates given that studies of neonatal pharmacokinetics suggest that the <60 min time frame for prophylactic dosing would also be effective [25].

**Table:** 

*Recommendation:*	**Administer appropriate preoperative antibiotic prophylaxis within 60** **min prior to skin incision**
*Evidence Quality:*	Low
*Recommendation Strength:*	Weak

#### Duration of postoperative antibiotics

Few studies investigate the optimal duration of perioperative antibiotic prophylaxis after neonatal intestinal surgery. Low quality studies show no difference in neonatal SSI rates when prophylactic antibiotics were given for less than 24 h as compared to greater than 24 h [26, 27]. Although adult literature demonstrates that a single preoperative dose of antibiotics is generally sufficient for prophylaxis, similar evidence in neonates is lacking. Given the significantly higher rate and severity of neonatal SSIs, general practice has been to provide longer periods of prophylactic antibiotics as demonstrated in a survey study where patients received inconsistent and prolonged prophylactic antibiotic courses with some extending beyond 1 week [26–28]. Antibiotic administration, however, carries an increased risk of invasive Candida infections, and emergence of resistant organisms [29]. In the setting of a documented infection or wound contamination, an appropriate therapeutic antibiotic regimen should be pursued.

**Table:** 

*Recommendation:*	**Discontinue postoperative antibiotics within 24** **h of surgery, unless ongoing treatment is required**
*Evidence Quality:*	Low
*Recommendation Strength:*	Weak

#### Preventing intraoperative hypothermia

Neonates are at high risk of surgical hypothermia (<36.5 °C), and temperature monitoring is frequently neglected [30]. Hypothermic infants suffer more respiratory adverse events and require more interventions than their non-hypothermic counterparts [31]. Neonates are at greatest risk of hypothermia in the OR [31]. Implementation of hypothermia bundles can significantly reduce perioperative hypothermia [32, 33]. Despite low quality of evidence, the risks of hypothermia in neonates warrant a strong recommendation for monitoring and pre-emptive measures to maintain normothermia.

**Table:** 

*Recommendation:*	**Continuously monitor intraoperative core temperature and take pre-emptive measures to prevent hypothermia (<36.5** °C) **and maintain normothermia**
*Evidence Quality:*	Low
*Recommendation Strength:*	Strong

#### Perioperative fluid management

Perioperative fluid management in neonates aims to maintain tissue perfusion, metabolic function, and acid–base-electrolyte status. Monitoring clinical response to fluids, blood glucose, blood gases and electrolytes is a key part of intraoperative care [34]. Isotonic solutions with glucose are recommended for intraoperative fluid administration. Both hyperglycema and hypoglycemia have been documented in neonates with different fluid regimens in the OR [35]. Glucose-containing fluids may decrease intraoperative hypoglycemia, but high concentrations may contribute to hyperglycemia [35]. Targeting glucose to 3.3– 7 mmol/L reflects definitions of hypo- and hyperglycemia in neonates [36, 37]. Targets for anesthetized infants, however, do not exist. Clinicians may target a slightly higher range for these infants, recognizing that levels above 8 mmol/L may have detrimental effects on neurodevelopment. Hypotonic IV fluids should not be used as they heighten the risk for hyponatremia [38]. Colloids are only recommended to recover normovolemia when crystalloids alone are not sufficient and blood products are not indicated. Despite moderate quality evidence supporting this recommendation, there are few studies demonstrating downstream effects. Therefore, specific regimens cannot be suggested and the strength of the recommendation is weak.

**Table:** 

*Recommendation:*	**Use perioperative fluid management to maintain tissue perfusion and prevent hypovolemia, fluid overload, hyponatremia, and hyperglycemia**
*Evidence Quality:*	Moderate
*Recommendation Strength:*	Weak

### Perioperative analgesia

#### Acetaminophen

Acetaminophen is an important part of a multimodal regime to limit opioid exposure after neonatal surgery. Multiple high-quality studies indicate that IV acetaminophen reduces postoperative morphine consumption in neonates when compared with other regimens [39]. When IV acetaminophen is not available, rectal acetaminophen should be given, although it may be less effective. Despite concerns about the hepatic effects of IV acetaminophen, low doses are well tolerated in term neonates and have a good safety profile when used for limited periods [40]. Although current evidence suggests that the short-term use of acetaminophen is safe, longer-term safety is less clear [40]. Acetaminophen should be given regularly (not prn) in postoperative neonates with strict adherence to the recommended dose, dosing interval, and maximum allowable daily dose.

**Table:** 

*Recommendation:*	**Unless contraindicated, administer acetaminophen regularly during the early postoperative period (not on an “as needed” basis) to minimize opioid use**
*Evidence Quality:*	High
*Recommendation Strength:*	Strong

#### Opioid use

Morphine is effective in treating postoperative pain following neonatal surgery [41]. However, pharmacokinetic differences lead to less predictable clinical effects in neonates as compared to older children with an increased variability in plasma concentrations of morphine and its metabolites [41]. Reduced doses and increased dosing intervals are necessary to avoid accumulation and the risk of sedation and respiratory depression [41].

With neonatal morphine use, the therapeutic window between analgesia and respiratory depression is narrow [41]. Other important adverse effects include hypotension and decreased gastrointestinal motility [42].

Moderate evidence supports the use of an opioid sparing, multimodal analgesia strategy in postoperative neonates. The lowest dose of opioid should be given for the shortest possible time. All neonates receiving opioids should be managed with continuous pulse oximetry, monitoring of other vital signs, and regular assessment of pain scores.

**Table:** 

*Recommendation:*	**Use an opioid-limiting strategy is recommended in the postoperative period. Manage breakthrough pain with the lowest effective dose of opioid with continuous monitoring**
*Evidence Quality:*	Moderate
*Recommendation Strength:*	Strong

#### Standard analgesia protocol

High-quality evidence demonstrates that ultrasound-guided regional anesthetic techniques and regular acetaminophen reduces the exposure of infants to opioids and other anesthetic agents [43]. Epidural analgesia, when combined with general anesthesia, decreases respiratory complications and shortens the time to bowel function [44, 45]. Regional blocks avoid some of the risks of epidurals while achieving good pain control [46]. In appropriate neonates, the use of regional anesthesia and regular (not prn) acetaminophen is recommended and may reduce the need for perioperative narcotics.

**Table:** 

*Recommendation:*	**Use regional anesthesia and acetaminophen perioperatively in combination with general anesthesia. Multimodal strategies including regional techniques should be continued postoperatively**
*Evidence Quality:*	High
*Recommendation Strength:*	Strong

#### Lingual sucrose/dextrose

Many high-quality studies have demonstrated the efficacy of lingual sucrose/dextrose as an analgesic in neonates. A large systematic review demonstrated the benefits of oral sucrose in neonates undergoing heel lance, venipuncture, and intramuscular injections [47]. A smaller number of studies showed sucrose to be of benefit for other interventions such as naso/orogastric tube insertion, with lower pain scores when compared with placebo [48]. Due to the low morbidity and high feasibility, we recommend lingual sucrose/dextrose to reduce pain during naso/orogastric tube placement and other minor painful procedures.

**Table:** 

*Recommendation:*	**Provide lingual sucrose/dextrose to reduce pain during naso/orogastric tube placement and other minor painful procedures**
*Evidence Quality:*	High
*Recommendation Strength:*	Strong

#### Optimal hemoglobin

Neonates require a distinct set of hemoglobin thresholds and transfusion guidelines. Neonates have a limited ability to tolerate stress, and anemia is associated with a high risk of mortality [49]. Due to the lack of a universally accepted definition of anemia in neonates, determining an optimal hemoglobin threshold is challenging. Most evidence-based sources recommend a restrictive hemoglobin threshold in term neonates given that no differences in short term outcomes have been found comparing restrictive and liberal strategies [49, 50]. Methods to decrease blood loss should be pursued, including measures to minimize blood sampling. When indicated, red blood cell transfusions should be single donor, leukocyte depleted, irradiated, and fresh [50]. Recommendations for the optimal hemoglobin threshold for term neonates ARE currently based on low quality evidence.

**Table:** 

*Recommendation:*	**Restrict transfusions to maintaining HgB≥90 (9** **g/dL for a term neonate with no oxygen requirement. Term neonates within the first week of life, intubated or with an oxygen requirement should be transfused to maintain a HgB≥110 (11** **g/dL)**
*Evidence Quality:*	Low
*Recommendation Strength:*	Weak

**Table:** 

*Recommendation:*	**Use written transfusion guidelines and take into account not only a target hemoglobin threshold, but also the clinical status of the neonate and local practices**
*Evidence Quality:*	Low
*Recommendation Strength:*	Weak

### Perioperative communication

Standardized perioperative communication and care processes can reduce adverse patient outcomes, ensure continuity of care and improve staff communication [51, 52]. A systematic review of postoperative handovers found that successful elements include: (a) checklist use (b) completion of urgent tasks prior to handover, (c) minimizing handover interruptions, (d) presence of all relevant team members, and (e) team communication training [53]. Staff engagement and teamwork are the most important factors for promoting a safe surgical environment [54]. Due to the potential adverse effects of miscommunication, complete interdisciplinary team participation in structured perioperative communication processes should be implemented [54].

**Table:** 

*Recommendation:*	**Implement perioperative multidisciplinary team communication with a structured process and protocol (“pre- and postoperative huddle”) utilizing established checklists**
*Evidence Quality:*	Moderate
*Recommendation Strength:*	Strong

### Parental involvement

Improving communication with parents by providing information on communication, family centered rounds, and using technology (e.g., smartphone texts) all have demonstrated improved patient outcomes and family satisfaction [55–58]. Parental involvement should be individualized, and special consideration provided for patients of different ethnicities, ages and genders [55, 56].

The discharge experience is frequently perceived by parents as confusing with inconsistent communication [57]. To better prepare parents for discharge after surgery, teaching necessary skills should be initiated early and continued throughout hospitalization [58, 59]. Providing educational opportunities increases parental knowledge, confidence, and satisfaction; and may improve infant developmental outcomes, increase compliance with well-baby checks, and reduce emergency room visits [59–61]. Written materials, audiovisual aids, and simulation have all been found to be helpful by parents [58, 59, 61, 62].

**Table:** 

*Recommendation:*	**Facilitate hands on care and purposeful practice by parents that is individualized to meet the unique needs of parents early during the admission. Sustain these to build the knowledge and skills of parents to take on a leading role as caregivers and facilitate their readiness for discharge**
*Evidence Quality:*	High
*Recommendation Strength:*	Strong

### Postoperative nutrition

#### Early feeding

There is high-quality evidence to support early enteral feeding in post-surgical neonates. Neonates that were fed early have a shorter LOS, and decreased SSIs with no increase in anastomotic leaks [63, 64]. Additionally, in very low birth weight infants, the early introduction of low volume feeds showed no increased incidence of NEC and a decreased incidence of sepsis [65, 66]. Although high-quality evidence supports this recommendation, variable clinical situations may require a delay in feeding so the recommendation was determined to be weak.

**Table:** 

*Recommendation:*	**Start early enteral feeds within 24–48** **h after surgery when possible. Do not wait for formal return of bowel function**
*Evidence Quality:*	High
*Recommendation Strength:*	Weak

#### Breast milk as first nutrition

Breast milk is a resource-friendly feeding choice for term infants, with a high level of evidence for its benefits. In post-surgical patients, where feeding intolerances are common, breast milk is typically well tolerated and the presence of immunoglobulin, prebiotics, and growth factors improve intestinal adaptation [67, 68]. The protective effect of breast milk on the development and recurrence of NEC in preterm and low birth weight infants is well described [69]. Breast milk consumption promotes development of beneficial fecal flora and suppresses growth of potential pathogenic organisms in term infants [70].

**Table:** 

*Recommendation:*	**Use breast milk as the first choice for nutrition**
*Evidence Quality:*	High
*Recommendation Strength:*	Strong

#### Urinary sodium monitoring

Infants with stomas commonly suffer from sodium depletion [71, 72]. Inadequate urine sodium concentration is associated with slower weight gain in infants undergoing intestinal surgery [72]. Neonates with stomas should undergo urinary sodium level monitoring. Sodium supplementation to maintain urinary sodium >30 mmol/L improves overall growth in surgical neonates [71]. The low quality of evidence supports a case-by-case approach to sodium supplementation.

**Table:** 

*Recommendation:*	**Monitor urinary sodium in all neonates with a stoma. Target urinary sodium should be greater than 30** **mmol/L and exceed the level of urinary potassium**
*Evidence Quality:*	Low
*Recommendation Strength:*	Weak

#### Mucous fistula refeeding

In neonates with stomas, mucous fistula refeeding can augment absorption of enteral nutrition required for healing and growth. Low to moderate quality studies of mucous fistula refeeding demonstrate improved weight gain, shorter duration of parenteral nutrition, decreased anastomotic leakage, and less cholestasis [73–75]. Complications are rare; however, the risk of bowel perforation and death lends caution [74]. The decision to proceed with mucous fistula feeding should include careful patient selection (term infants with a healthy mucous fistula and distal small bowel), ongoing monitoring, and nursing education.

**Table:** 

*Recommendation:*	**Use mucous fistula refeeding in neonates with enterostomy to improve growth**
*Evidence Quality:*	Moderate
*Recommendation Strength:*	Weak

### Excluded items and weak evidence

In some instances, good quality data were not available (urinary catheter use) or data were conflicting (chlorhexidine vs. povidone iodine based skin preparation) preventing the development of recommendations.

Many recommendations within this guideline are supported by weak evidence. In some cases, supporting evidence is inferred from high-quality studies in other populations (preoperative antibiotics); in other cases, potential benefits were judged to outweigh harms (prevention of hypothermia). Finally, in some cases, weak evidence was bolstered by national standards (transfusions) [50].

## Conclusion

ERAS^®^ guidelines integrate evidence-based practices into multimodal care pathways to optimize postoperative recovery [1]. Due to neonatal physiological differences and the unique nature of the care team, a neonatal ERAS^®^ guideline is necessarily different from adult guidelines. This article presents the evidence base for 17 ERAS^®^ recommendations.

Often the data supporting recommendations are of low to moderate quality. The guideline will undergo regular review, and higher quality data will be used to further improve recommendations [2]. Effective implementation requires local leadership and consideration of local context. We anticipate that a longitudinal, iterative approach to developing neonatal ERAS^®^ guidelines will continue to improve the care of neonatal surgical patients.

## Electronic supplementary material

Below is the link to the electronic supplementary material.

Online Resource 1 Topics identified by guideline committee for ERAS^®^ recommendation development (DOCX 14 kb)

Online Resource 2 Systematic search strategies used for each topic (DOCX 43 kb)

Online Resource 3 PRISMA diagrams outlining evidence screening process (DOCX 245 kb)

Online Resource 4 Summary of evidence and quality of evidence used in support of each recommendation (DOCX 120 kb)

Online Resource 5 Complete guideline document with full reference list (DOCX 184 kb)
