# Supplementary material for: Consensus Guidelines for Perioperative Care in Neonatal Intestinal Surgery: Enhanced Recovery After Surgery (ERAS®) Society Recommendations
Source: World J Surg. 2020 May 8;44(8):2482–92. doi: 10.1007/s00268-020-05530-1 (PMC7326795; doi:10.1007/s00268-020-05530-1)
Supplement: Supplementary file 1 — Topics identified by guideline committee for ERAS® recommendation development (DOCX 14 kb) [file 268_2020_5530_MOESM1_ESM.docx]

Online Resource 1 – Study Topics

**Table 1.** Topics identified by guideline committee for ERAS® recommendation development

| # | **Topic** |
| --- | --- |
| 1 | Temperature regulation/prevention of intraoperative hypothermia |
| 2 | Standard anesthetic protocol and fluid management |
| 3 | Surgical practices |
| 4 | Optimal hemoglobin level |
| 5 | Postoperative analgesia |
| 6 | Parental involvement |
| 7 | Management of transitional circulation |
| 8 | Urinary drainage |
| 9 | Postoperative skin/stoma care |
| 10 | Perioperative communication and team structure |
| 11 | Postoperative vomiting with nasogastric intubation |
| 12 | Role of physiotherapy and occupational therapy |
| 13 | Postoperative nutritional care |
| 14 | Antimicrobial prophylaxis and skin preparation |
