# Supplementary material for: Consensus Guidelines for Perioperative Care in Neonatal Intestinal Surgery: Enhanced Recovery After Surgery (ERAS®) Society Recommendations
Source: World J Surg. 2020 May 8;44(8):2482–92. doi: 10.1007/s00268-020-05530-1 (PMC7326795; doi:10.1007/s00268-020-05530-1)
Supplement: Supplementary file 2 — Systematic search strategies used for each topic (DOCX 43 kb) [file 268_2020_5530_MOESM2_ESM.docx]

Online Resource 2 – Systematic Search Strategies used for each Topic

**Table 1.** MEDLINE search strategy for temperature regulation/prevention of intraoperative hypothermia

| **Population** | **Procedure** | **Topic** |
| --- | --- | --- |
| Neonatology.mp  Neonatology/  Neonat*.mp.  Infant/  Infant, Newborn/  Infant*.mp.  Term birth.mp.  Term Birth/  Gestational age.mp.  Gestational Age/  (“37”adj2week*).mp. | General Surgery/  Surgery.mp.  Surgical Procedures, Operative | Body temperature regulation.mp.  Body Temperature Regulation/  Thermoregulation.mp  (Temperature adj2 regulation).mp.  (Climate adj2 regulation).mp.  (Temperature adj2 control).mp.  (Temperature adj2 management).mp.  (Climate adj2 management).mp.  (Temperature adj2 super*).mp.  (Climate adj2 super*).mp.  (Temperature adj2 adjust*).mp.  (Climate adj2 adjust*).mp.  (Prevent* adj2 hyperthermia).mp.  (Prevent* adj2 hypothermia).mp.  (Avoid adj2 hyperthermia).mp.  (Avoid* adj2 hypothermia).mp. |

**Table 2.** MEDLINE search strategy for standard anesthetic protocol

| **Population** | **Procedure** | **Topic** |
| --- | --- | --- |
| Infant, Newborn/  Neonatology/  Neonat*.mp.  Gestational Age/  Term Birth/  (“37” adj2 week*).mp. | Anastomosis, surgical/  Digestive system surgical procedures/  Ostomy/ | Anesthesia/  Anesthetics/  Anesthetics.mp.  Anesthesia.mp.  Anesthetic agents.mp.  Anesthetic drugs.mp.  Spinal anesthesia .mp.  Anesthesia, Spinal/  General anesthesia.mp.  Anesthesia, General/  Epidural anesthesia.mp.  Anesthesia, Epidural/  Regional anesthesia.mp.  Anesthesia, Conduction/  Caudal Block .mp.  Anesthesia, Caudal/  Caudal Epidural.mp.  Anesthesia, Epidural/  Lumbar epidural.mp.  Anesthetics, Local/ |

**Table 3.** MEDLINE search strategy for fluid management

| **Population** | **Procedure** | **Topic** |
| --- | --- | --- |
| Infant, Newborn/  Neonatology/  Neonat*.mp.  Gestational Age/  Term Birth/  (“37” adj2 week*).mp. | Anastomosis, surgical/  Digestive system surgical procedures/  Ostomy/ | Fluid Therapy.mp.  Fluid Therapy/  Rehydration.mp.  Fluid therap*.mp  Fluids.mp.  Intraoperative Fluids.mp.  Postoperative Fluids.mp.  Fluid Management.mp  Crystalloid.mp.  Colloid.mp.  Colloids/  Intraoperative Care/  Hyponatremia/  Dilutional Hyponatremia.mp.  Infusions.mp.  Infusions, Intravenous/ |

**Table 4.** MEDLINE search strategy for surgical practices

| **Population** | **Procedure** | **Topic** |
| --- | --- | --- |
| Infant, Newborn/  Neonatology/  Neonat*.mp.  Gestational Age/  Term Birth/  (“37” adj2 week*).mp. | (Intestin* adj2 resection*).mp.  Intestinal resection surgery.mp.  Intestinal resections.mp  Anastomosis, surgical/  Digestive system surgical procedures/  Ostomy/  Bowel surgery.mp | Minimally Invasive Surgical Procedures/  Minimally invasive.mp.  Surgical practices.mp.  Surgical approach.mp.  Surgical technique.mp. |

**Table 5.** MEDLINE search strategy for optimal hemoglobin levels

| **Population** | **Procedure** | **Topic** |
| --- | --- | --- |
| Infant, Newborn/  Infant/  Infant.mp.  Neonatology.mp  Neonatology/  Neonat*.mp.  Gestational Age/  Term Birth/  Term birth.mp. | Anastomosis, surgical/  Digestive system surgical procedures/  Perioperative care/ | Hemoglobins/  Hemoglobin.mp  Iron/  Anema, Iron-Deficiency/  Ferrous Compounds/  Ferric Compounds/  Eryhem.mp.  Blood Transfusion/  Blood transfusion*.mp.  Erythropoietin/  Hematocrit.mp.  Hematocrit/ |

**Table 6.** MEDLINE search strategy for postoperative analgesia

| **Population** | **Procedure** | **Topic** |
| --- | --- | --- |
| Infant, Newborn/  Infant/  Infant.mp.  Neonatology.mp  Neonatology/  Neonat*.mp.  Gestational Age/  Term Birth/  Term birth.mp. | Anastomosis, surgical/  Digestive system surgical procedures/  Perioperative care/ | Analgesics, Opioid/  Pain Management/  Pain, Postoperative/  Analgesics/  Pain relief.mp.  Morphine.mp  Morphine/  Morphine Derivatives/  Fentanyl.mp.  Fentanyl/  Paracetamol.mp.  Acetaminophen/  NSAID.mp  Anti-Inflammatory Agents, Non-Steroidal/  Pain management.mp.  Pain Management/  Narcotics.mp.  Narcotics/  Ibuprofen.mp.  Ibuprofen/  Sucrose/  Sugar analgesia.mp.  Pain postoperative.mp. |

**Table 7.** CINAHL search strategy for postoperative analgesia

| **Population** | **Procedure** | **Topic** |
| --- | --- | --- |
| Term Birth/  Infant, Newborn Diseases+/  Gestational Age/  Infant, Postmature/  Infant, Newborn+/  Infant, Large for Gestational Age /  Infant, Small for Gestational Age/  Intensive Care Units, Neonatal/  Intensive Care, Neonatal/  Neonatal Intensive Care Nursing/  Neonat*  Infant  Newborn  Neonatology term birth  Gestational age  37 weeks old and surgery  Neonatal intensive care unit | Anastomosis, Surgical+/  Laparotomy/  Surgery, Digestive System+/  Surgery, Laparoscopic+/ Preoperative Period/ Ostomy+/  Perioperative care+/ | Postoperative Pain/  Pain Management/  Pain Management (Iowa NIC)/  Analgesia/  Patient-Controlled Analgesia/  Anesthesia and Analgesia/  Sucrose/  Ibuprofen/  Acetaminophen/  Narcotics/  Analgesics, Opioid/  Treatment Related Pain/ |

**Table 8.** MEDLINE search strategy for parental involvement

| **Population** | **Procedure** | **Topic** |
| --- | --- | --- |
| Infant, Newborn/  Infant/  Infant.mp.  Neonatology.mp  Neonatology/  Neonat*.mp.  Gestational Age/  Term Birth/  Term birth.mp.  Intensive care units neonatal.mp.  Intensive Care Units, Neonatal/ | Anastomosis, surgical/  Digestive system surgical procedures/  Perioperative care/ Perioperative period/  Ostomy/  Ostomy.mp.  Ileostomy/  Ileostomy.mp  Colectomy/  Colectomy.mp.  Bowel surgery.mp.  Intestinal Obstruction/  Intestinal resection surgery.mp. | Parents/  Parenting/  Parental involvement.mp  Parents/ed  Family care model.mp.  Family/  Patient Participation/  Parent* participation.mp.  Professional-Family Relations/  Professional-family relations.mp  Parent-Child Relations/  Parent-child relations.mp.  Patient Education as Topic/  Parent education.mp.  Stress, Psychological/px  Mothers/px  Depression/px  Anxiety/px  Colostomy/ed  Parent* engagement.mp.  Parent.mp. |

**Table 9.** CINAHL search strategy for parental involvement

| **Population** | **Procedure** | **Topic** |
| --- | --- | --- |
| Term Birth/  Infant, Newborn Diseases+/  Gestational Age/  Infant, Postmature/  Infant, Newborn+/  Infant, Large for Gestational Age /  Infant, Small for Gestational Age/  Intensive Care Units, Neonatal/  Intensive Care, Neonatal/  Neonatal Intensive Care Nursing/  Neonat*  Infant  Newborn  Neonatology term birth  Gestational age  37 weeks old and surgery  Neonatal intensive care unit | Anastomosis, Surgical+/  Laparotomy/  Surgery, Digestive System+/  Surgery, Laparoscopic+/ Preoperative Period/ Ostomy+/  Perioperative care+/ | Parent-Child Relations/  Parent Education: Childrearing Family (Iowa NIC)/  Parent Education: Childbearing Family (Iowa NIC)/  Parent-Infant Relations/  Parental Attitudes/  Biological Parents/  Single Parent/  Parental Behaviour/  Adolescent Parents/  Adoptive Parents/  Parental Role Conflict (Saba CCC)/  Parental Role Conflict (NANDA)/  Parent involvement  Family Centred Care  Developmental care  Parental counselling |

**Table 10.** MEDLINE search strategy for management of transitional circulation

| **Population** | **Procedure** | **Topic** |
| --- | --- | --- |
| Infant, Newborn/  Infant/  Infant.mp.  Neonatology.mp  Neonatology/  Neonat*.mp.  Gestational Age/  Term Birth/  Term birth.mp. | Anastomosis, surgical/  Digestive System Surgical Procedures/  Ostomy/  Ostomy.mp.  Stoma.mp.  Intestinal resection*.mp.  Colectomy/  Colon resection.mp.  Intestinal repair.mp.  Ileostomy.mp.  Ileostomy/  Bowel surgery.mp.  Laparoscopy/  Laparoscopic resection*.mp.  Laparotomy/ | Persistent pulmonary hypertension of the newborn.mp.  Hypertension, Pulmonary/  Persistent newborn pulmonary hypertension.mp.  (PPHN adj2 avoid*).mp  (avoid* adj2 pulmonary hypertension).mp.  (Avoid* adj2 hypoxia).mp.  (Avoid* adj2 hypothermia).mp.  (Avoid* adj2 acidosis).mp.  (Avoid* adj2 hypovolemia).mp.  (Avoid* adj2 hypertension).mp  (Avoid* adj2 stress).mp  (Avoid* adj2 pain).mp  (Prevent* adj2 hypoxia).mp.  (Prevent* adj2 hypothermia).mp.  (Prevent* adj2 acidosis).mp.  (Prevent* adj2 hypovolemia).mp.  (Prevent* adj2 hypertension).mp.  (Prevent* adj2 stress).mp.  (Prevent* adj2 pain).mp.  (Diagnos* adj2 pulmonary hypertension).mp.  (Manag* adj2 pulmonary hypertension).mp.  (Intervention* adj2 pulmonary hypertension).mp.  Hypertension, Pulmonary/  AND  Diagnosis, Differential/  Early Diagnosis/  Diagnosis-Related Groups/  Diagnosis/  Diagnosis, Computer-Assisted Diagnosis.mp  Treatment.mp  Therapeutics/  Intervention.mp  Manag*.mp. |

**Table 11.** MEDLINE search strategy for urinary drainage

| **Population** | **Procedure** | **Topic** |
| --- | --- | --- |
| Infant, Newborn/  Infant/  Infant.mp.  Neonatology.mp  Neonatology/  Neonat*.mp.  Gestational Age/  Term Birth/  Term birth.mp. | Anastomosis, surgical/  Digestive system surgical procedures/  Ostomy/  Laparotomy/ | Urinary drainage.mp.  Urinary emptying.mp.  Foley catheterization.mp.  Foley.mp.  Urinary Catheterization/  Catheterization/  Catheter.mp.  Catheters/ |

**Table 12.** CINAHL search strategy for urinary drainage

| **Population** | **Procedure** | **Topic** |
| --- | --- | --- |
| Term Birth/  Infant, Newborn Diseases+/  Gestational Age/  Infant, Postmature/  Infant, Newborn+/  Infant, Large for Gestational Age /  Infant, Small for Gestational Age/  Intensive Care Units, Neonatal/  Intensive Care, Neonatal/  Neonatal Intensive Care Nursing/  Neonat*  Infant  Newborn  Neonatology term birth  Gestational age  37 weeks old and surgery  Neonatal intensive care unit | Anastomosis, Surgical+/  Laparotomy/  Surgery, Digestive System+/  Surgery, Laparoscopic+/ Preoperative Period/ Ostomy+/  Perioperative care+/ | Urinary Catheterization+/  Urinary Catheterization, Intermittent/  Catheters, Urinary+/  Catheter Care, Urinary+/  Catheter Irrigation, Urinary/  Urinary Catheterization (Iowa NIC)+/  Urinary Catheterization: Intermittent (Iowa NIC)  Catheter Care, Suprapubic/  Antiinfective Agents, Urinary+/  Urinary Fistula+/ |

**Table 13.** MEDLINE search strategy for postoperative skin care/ stoma care

| **Population** | **Procedure** | **Topic** |
| --- | --- | --- |
| Infant, Newborn/  Infant/  Infant.mp.  Neonatology.mp  Neonatology/  Neonat*.mp.  Gestational Age/  Term Birth/  Term birth.mp. | Surgical procedures operative.mp.  Surgical Procedures, Operative/ | Skin Care/  (Stoma adj2 care).mp.  (Wound adj2 care).mp. |

**Table 14.** CINAHL search strategy for postoperative skin care/ stoma care

| **Population** | **Procedure** | **Topic** |
| --- | --- | --- |
| Term Birth/  Infant, Newborn Diseases+/  Gestational Age/  Infant, Postmature/  Infant, Newborn+/  Infant, Large for Gestational Age /  Infant, Small for Gestational Age/  Intensive Care Units, Neonatal/  Intensive Care, Neonatal/  Neonatal Intensive Care Nursing/  Neonat*  Infant  Newborn  Neonatology term birth  Gestational age  37 weeks old and surgery  Neonatal intensive care unit | Anastomosis, Surgical+/  Laparotomy/  Surgery, Digestive System+/  Surgery, Laparoscopic+/ Preoperative Period/ Ostomy+/  Perioperative care+/ | Wound Care (Iowa NIC)+/  Wound Care+/  Wound Care: Closed Drainage (Iowa NIC)/  Ostomy Care+/  Skin Care+/  Skin Care: Topical Treatments (Iowas NIC)/  Skin Care (Saba CCC)+/  Wound Care (Saba CCC)+/  Surgical Wound Care+/ |

**Table 15.** MEDLINE search strategy for perioperative communication and team structure

| **Population** | **Procedure** | **Topic** |
| --- | --- | --- |
| Infant, Newborn/  Infant/  Infant.mp.  Neonatology.mp  Neonatology/  Neonat*.mp.  Gestational Age/  Term Birth/  Term birth.mp.  Neonatal Intensive Care Unit*.mp.  Intensive Care Units, Neonatal/  NICU.mp. | Surgical Procedures, Operative/  Surgical procedures, operative.mp. | Interdisciplinary communication.mp.  Interdisciplinary Communication/  Interprofessional Relations/  Communication.mp.  Perioperative education.mp.  Preoperative education.mp.  Interdisciplinary.mp  Interprofessional relations.mp.  Patient Care Team/  AND  Communication/  Cooperative Behaviour/  Efficiency, Organizational/  **_______________________________**  Nursing, Team AND Efficiency, Organizational/  Communication/ AND Nursing Staff/ |

**Table 16.** CINAHL search strategy for perioperative communication and team structure

| **Population** | **Procedure** | **Topic** |
| --- | --- | --- |
| Term Birth/  Infant, Newborn Diseases+/  Gestational Age/  Infant, Postmature/  Infant, Newborn+/  Infant, Large for Gestational Age /  Infant, Small for Gestational Age/  Intensive Care Units, Neonatal/  Intensive Care, Neonatal/  Neonatal Intensive Care Nursing/  Neonat*  Infant  Newborn  Neonatology term birth  Gestational age  37 weeks old and surgery  Neonatal intensive care unit | Anastomosis, Surgical+/  Laparotomy/  Surgery, Digestive System+/  Surgery, Laparoscopic+/ Preoperative Period/ Ostomy+/  Perioperative care+/ | Multidisciplinary Care Team+/  Attitude of Health Personnel+/  Organizational Culture+  Organizational Objectives/  Organizational Theory/  Organizational Change/  Nonverbal Communication/  Communication Protocols+/  Communication Skills Training/  Communication Skills/  Communication Barriers/  Communication Care (Saba CCC)/  Communication Impairment (Saba CCC)+/  Cooperative Behavior/ |

**Table 17.** MEDLINE search strategy for postoperative vomiting with nasogastric intubation

| **Population** | **Procedure** | **Topic** |
| --- | --- | --- |
| Infant, Newborn/  Infant/  Infant.mp.  Neonatology.mp  Neonatology/  Neonat*.mp.  Gestational Age/  Term Birth/  Term birth.mp.  Intensive care unit neonatal.mp.  Intensive Care Units, Neonatal/ | Surgical Procedures, Operative/  Surgical procedures, operative.mp.  Surgery.mp.  General Surgery/ | Vomit*.mp.  Vomiting/  Emesis.mp.  Regurgitation.mp.  AND  Intubation.mp.  Intubation/  Intubation, Gastrointestinal/  (Nasogastric adj2 intubation).mp. |

**Table 18.** CINAHL search strategy for postoperative vomiting with nasogastric intubation

| **Population** | **Procedure** | **Topic** |
| --- | --- | --- |
| Term Birth/  Infant, Newborn Diseases+/  Gestational Age/  Infant, Postmature/  Infant, Newborn+/  Infant, Large for Gestational Age /  Infant, Small for Gestational Age/  Intensive Care Units, Neonatal/  Intensive Care, Neonatal/  Neonatal Intensive Care Nursing/  Neonat*  Infant  Newborn  Neonatology term birth  Gestational age  37 weeks old and surgery  Neonatal intensive care unit | Anastomosis, Surgical+/  Laparotomy/  Surgery, Digestive System+/  Surgery, Laparoscopic+/ Preoperative Period/ Ostomy+/  Perioperative care+/ | Vomiting+/  Nausea and Vomiting+/  Anticipatory Nausea and Vomiting/  Vomiting (Saba CCC)/  Gastroesophageal Reflux/  Vomit*  Emesis  Regurgitation  Vomiting  AND  Intubation, Gastrointestinal/  Gastrointestinal Intubation (Iowa NIC)/ |

**Table 19.** MEDLINE search strategy for role of Physiotherapy and Occupational Therapy

| **Population** | **Procedure** | **Topic** |
| --- | --- | --- |
| Infant, Newborn/  Infant/  Infant.mp.  Neonatology.mp  Neonatology/  Neonat*.mp.  Gestational Age/  Term Birth/  Term birth.mp.  Intensive care unit neonatal.mp.  Intensive Care Units, Neonatal/ | Anastomosis, surgical/  Digestive system surgical procedures/  Ostomy/  Laparotomy/  Perioperative Care/  Perioperative Period/ | Occupational Therapy/  Occupational therapy.mp.  Physiotherapy.mp.  Physical Therapy Modalities/  Rehabilitation/  Physical and Rehabilitation Medicine/  Rehabilitation.mp.  Mobilization.mp. |

**Table 20.** CINAHL search strategy for role of Physiotherapy and Occupational therapy

| **Population** | **Procedure** | **Topic** |
| --- | --- | --- |
| Term Birth/  Infant, Newborn Diseases+/  Gestational Age/  Infant, Postmature/  Infant, Newborn+/  Infant, Large for Gestational Age /  Infant, Small for Gestational Age/  Intensive Care Units, Neonatal/  Intensive Care, Neonatal/  Neonatal Intensive Care Nursing/  Neonat*  Infant  Newborn  Neonatology term birth  Gestational age  37 weeks old and surgery  Neonatal intensive care unit | Anastomosis, Surgical+/  Laparotomy/  Surgery, Digestive System+/  Surgery, Laparoscopic+/ Preoperative Period/ Ostomy+/  Perioperative care+/ | Physical Therapy/  Pediatric Physical Therapy/  Physical Therapy Practice, Research-Based/  Physical Therapy Practice, Evidence-Based/  Canadian Physiotherapy Association/  Pediatric Occupational Therapy/  Occupational Therapy Assistants/  Combined Modality Therapy+  Research, Occupational Therapy/  Occupational Therapy Practice, Research-Based/  Occupational Therapy Practice, Evidence-Based/  Occupational Therapy Assessment/  Occupational therapy  Physiotherapy  Rehabilitation  Mobilization  Physical therapy modalities |

**Table 21.** MEDLINE search strategy for postoperative nutritional care

| **Population** | **Procedure** | **Topic** |
| --- | --- | --- |
| Term birth.mp.  Term Birth/  Gestational Age/  Infant, Newborn/  Neonatology.mp.  Neonatalogy/  Neonat*.mp. | Colostomy.mp.  Colostomy/  Anastomosis, surgical/  Digestive system surgical procedures/  Laparotomy/  Ostomy/  Ileostomy.mp  Ileostomy/  Colectomy.mp  Colectomy/  Bowel surgery.mp  Intestinal obstruction.mp.  Intestinal Obstruction/ | Enteral Nutrition/  Feeding Behaviour/  Timing of feeding.mp  Advancing feeding.mp  Feed* progression.mp  Continuous feed*.mp  Bolus feed*.mp  Weight Gain/  Body Mass Index/  Optimal weight gain.mp  Growth chart.mp  Growth Charts/  Optimal growth requirements.mp  Nutrition assessment.mp  Nutrition Assessment/  Nutritional Status/  Adequate nutrition.mp  Breast Feeding/  Bottle Feeding/  Breastmilk feeding.mp  Infant Food/  Infant Formula/  Formula.mp  Elemental formula.mp  Semi elemental formula.mp  Tact formula.mp  Tube feeding.mp  Oral feeding.mp  Introduction of feeds.mp  Nutritional requirements.mp  Nutritional Requirements/ |

**Table 22.** CINAHL search strategy for postoperative nutritional care

| **Population** | **Procedure** | **Topic** |
| --- | --- | --- |
| Term Birth/  Infant, Newborn Diseases+/  Gestational Age/  Infant, Postmature/  Infant, Newborn+/  Infant, Large for Gestational Age /  Infant, Small for Gestational Age/  Intensive Care Units, Neonatal/  Intensive Care, Neonatal/  Neonatal Intensive Care Nursing/  Neonat*  Infant  Newborn  Neonatology term birth  Gestational age  37 weeks old and surgery  Neonatal intensive care unit | Anastomosis, Surgical+/  Laparotomy/  Surgery, Digestive System+/  Surgery, Laparoscopic+/ Preoperative Period/ Ostomy+/  Perioperative care+/ | Infant Feeding, Supplemental/  Infant Feeding Schedules/  Feeding Tube Irrigation/  Infant Feeding/  Feeding Tubes/  Timing of feeding  Enteral Feeding Pumps/  Feeding Tube Care/  Introduction of feeding  Advancing feeding  Feeding Methods/  Enteral Nutrition/  Enteral nutrition  Enteral Feeding (Saba CCC)  Enteral Tube Feeding (Iowa NIC)  Enteral feeding  Oral feeding  Oral vs tube feeding  Tube feeding  Elemental formula  Infant Formula/  Semi-elemental formula  In-tact formula infant  Continuous feeding  Bolus feeding  Ineffective Infant Feeding Pattern (NANDA)/  Infant Nutritional Physiology/  Method of infant feeding  Nutritional Assessment/  Nutritional Physiology/  Nutrition assessment  Optimal growth  Optimal nutrition  Nutritional Requirements/  Nutritive Value/  Adequate nutrition  Nutritional intake |

**Table 23.** MEDLINE search strategy for antimicrobial prophylaxis and skin preparation

| **Population** | **Procedure** | **Topic** |
| --- | --- | --- |
| Term birth.mp.  Term Birth/  Gestational Age/  Infant, Newborn/  Infant.mp.  Infant/  Neonatology.mp.  Neonatalogy/  Neonat*.mp.  Intensive care unit neonatal.mp.  Intensive Care Units, Neonatal/ | Anastomosis**,** Surgical/  Digestive System Surgical Procedures/  Laparotomy/  Ostomy/  Perioperative Care/  Perioperative Period/ | Antibiotic prophylaxis/  Antimicrobial prophylaxis.mp.  Antimicrobial prophylaxis.mp.  (Antibiotic adj2 premedication).mp.  Anti-Bacterial Agents/  Antibacterial agent*.mp  Skin preparation.mp  Anti-Infective Agents, Local/  Povidone/  Poviodone.mp  Povidone-Iodine/  Chlorhexidine/ |

**Table 24.** CINAHL search strategy for antimicrobial prophylaxis and skin preparation

| **Population** | **Procedure** | **Topic** |
| --- | --- | --- |
| Term Birth/  Infant, Newborn Diseases+/  Gestational Age/  Infant, Postmature/  Infant, Newborn+/  Infant, Large for Gestational Age /  Infant, Small for Gestational Age/  Intensive Care Units, Neonatal/  Intensive Care, Neonatal/  Neonatal Intensive Care Nursing/  Neonat*  Infant  Newborn  Neonatology term birth  Gestational age  37 weeks old and surgery  Neonatal intensive care unit | Anastomosis, Surgical+/  Laparotomy/  Surgery, Digestive System+/  Surgery, Laparoscopic+/ Preoperative Period/ Ostomy+/  Perioperative care+/ | Surgical Wound Infection/  Antiinfective Agents+/  Antiinfective Agents, Fluroquinolone+/  Antiinfective Agents, Local+/  Antiinflammatory Agents, Topical+/  Antiinfective Agents, Quinolone+/  Antibiotic Prophylaxis/  Antibiotic prophylaxis  Antimicrobial prophylaxis  Antibiotic premedication  Antibiotic  Antibacterial agent  Skin preparation  Antiseptic  Anti-infective agents  Topical anti-infective agents  Povidone  Povidone-iodine  Iodine/  Povidone+/  Iodine Compounds+  Skin Preparation, Surgical/ |

**Table 25.** Additional MEDLINE search for parental involvement

| **Setting** | **Population** | **Topic** |
| --- | --- | --- |
| Intensive Care Units, Neonatal/ | Parents/ | Engagement.mp  Involvement.mp  Education/ |

**Table 26.** Additional MEDLINE search for communication

| **Setting** | **Population** | **Topic** |
| --- | --- | --- |
| Intensive Care Units, Neonatal/ | Nursing Staff, Hospital  Parents/  Physicians/  Professional-Family Relations/  Patient Care Team/ | Communication/ |

**Table 27.** Additional MEDLINE search for team structure

| **Setting** | **Topic** |
| --- | --- |
| Intensive Care Units, Neonatal/ma, mt, og, st  Intensive Care, Neonatal/ma, mt, og, st | Cooperative Behaviour |

**Table 28.** Additional MEDLINE search for transitional circulation

| **Population** | **Topic** |
| --- | --- |
| Infant, Newborn  Infant, newborn.mp | Persistent Fetal Circulation Syndrome/th |

**Table 29.** Additional Google Scholar Searches

| **Topic** | **Search words** | **Date Performed** | **Pages Reviewed** |
| --- | --- | --- | --- |
| Perioperative Nutrition | Early feed postoperative neonate | January 22^nd^, 2019 | Pages 1-3 |
|  | Breast milk surgery neonate |  |  |
|  | Breast milk neonate |  |  |
| Postoperative Analgesia | Sucrose pain neonate | March 19^th^, 2019 | Pages 1-3 |
|  | Sucrose pain surgical neonates | May 1^st^, 2019 |  |
|  | Sucrose procedural pain neonates |  |  |
