# Supplementary material for: Consensus Guidelines for Perioperative Care in Neonatal Intestinal Surgery: Enhanced Recovery After Surgery (ERAS®) Society Recommendations
Source: World J Surg. 2020 May 8;44(8):2482–92. doi: 10.1007/s00268-020-05530-1 (PMC7326795; doi:10.1007/s00268-020-05530-1)
Supplement: Supplementary file 3 — PRISMA diagrams outlining evidence screening process (DOCX 245 kb) [file 268_2020_5530_MOESM3_ESM.docx]

Online Resource 3 – PRISMA Diagrams Outlining Evidence Screening Process

**PRISMA Diagram 1:** Surgical practices

Records identified through database search (120)

Full papers available for screening (50)

Excluded abstracts (487)

Full paper assessment for inclusion (47)

Excluded full papers (19)

Full papers included for analysis (28)

Additional PubMed search (417)

**PRISMA Diagram 2:** Antimicrobial prophylaxis and skin preparation

Records identified through database search (211)

Full papers available for screening (7)

Excluded abstracts (204)

Full paper assessment for inclusion (35)

Excluded full papers (18)

Additional searches (3)

Reference snowballing (25)

Full papers included for analysis

Antimicrobial Prophylaxis (9)

Skin Preparation (8)

Other: Guidelines, Protocols, and Surveys

- Antimicrobial Prophylaxis
  - Google Search: Guidelines = 6; Survey = 1
- Skin Preparation
  - Google Search: Guidelines = 6; Survey = 1

**PRISMA Diagram 3:** Nasogastric tubes

Records identified through database search (103)

Full papers available for screening (10)

Excluded abstracts (93)

Full paper assessment for inclusion (19)

Excluded full papers (7)

Full papers included for analysis (12)

Reference snowballing (7)

Additional searches (2)

**PRISMA Diagram 4:** Role of occupational therapy/physiotherapy

Records identified through database search (138)

Full papers available for screening (6)

Excluded abstracts (132)

Full paper assessment for inclusion (12)

Excluded full papers (6)

Full papers included for analysis (6)

Reference snowballing (6)

**PRISMA Diagram 5:** Anesthesia

Records identified through database search (349)

Full papers available for screening (38)

Excluded abstracts (311)

Full paper assessment for inclusion (53)

Excluded full papers (29)

Reference snowballing (10)

Full papers included for analysis

Anesthetic Protocol (13)

Fluid Management (4)

Temperature Management (7)

Additional searches (5)

Other: Guidelines, Protocols, and Surveys

- Anesthesia
  - Google Search: Guidelines = 3

**PRISMA Diagram 6:** Urinary drainage

Records identified through database search (118)

Full papers available for screening (3)

Excluded abstracts (115)

Full paper assessment for inclusion (3)

Excluded full papers (3)

Full papers included for analysis (0)

Additional searches (0)

**PRISMA Diagram 7:** Optimal hemoglobin

Records identified through database search (82)

Full papers available for screening (5)

Excluded abstracts (77)

Full papers included for analysis (5)

Additional searches (2)

Expert identification (4)

Full paper assessment for inclusion (11)

Excluded full papers (6)

**PRISMA Diagram 8:** Postoperative analgesia

Records identified through database search (263)

Full papers available for screening (15)

Excluded abstracts (248)

Full paper assessment for inclusion (50)

Excluded full papers (20)

Full papers included for analysis (30)

Additional searches (31)

Reference snowballing (4)

Other: Guidelines, Protocols, and Surveys

- Postoperative Analgesia
  - Google Search: Guidelines = 4

**PRISMA Diagram 9:** Parental involvement and perioperative communication

Records identified through database search (724)

Full Papers available for screening (163)

Excluded abstracts (561)

Full paper assessment for inclusion (166)

Excluded full papers (132)

Reference snowballing (3)

Full papers included for analysis

Parental involvement (22)

Communication (12)

**PRISMA Diagram 10:** Perioperative nutrition

Records identified through database search (359)

Full Papers available for screening (32)

Excluded abstracts (327)

Full paper assessment for inclusion (67)

Excluded full papers (44)

Full papers included for analysis (23)

Additional searches (20)

Reference snowballing (15)

Other: Guidelines, Protocols, and Surveys

- Postoperative Analgesia
  - Google Search: Guidelines = 7

**PRISMA Diagram 11:** Stoma care

Records identified through database search (143)

Full Papers available for screening (13)

Excluded abstracts (130)

Full paper assessment for inclusion (21)

Excluded full papers (18)

Full papers included for analysis (3)

Reference snowballing (5)

Additional searches (3)

Other: Guidelines, Protocols, and Surveys

- Stoma Care
  - Google Search: Guidelines = 10

**PRISMA Diagram 12:** Transitional circulation

Records identified through database search (405)

Full Papers available for screening (200)

Excluded abstracts (205)

Full paper assessment for inclusion (205)

Excluded full papers (189)

Full papers included for analysis (16)

Additional searches (5)
