# Supplementary material for: Consensus Guidelines for Perioperative Care in Neonatal Intestinal Surgery: Enhanced Recovery After Surgery (ERAS®) Society Recommendations
Source: World J Surg. 2020 May 8;44(8):2482–92. doi: 10.1007/s00268-020-05530-1 (PMC7326795; doi:10.1007/s00268-020-05530-1)
Supplement: Supplementary file 4 — Summary of evidence and quality of evidence used in support of each recommendation (DOCX 120 kb) [file 268_2020_5530_MOESM4_ESM.docx]

Online Resource 4 – Summary of Evidence and Quality of Evidence Used in Support of each Recommendation

**Primary Anastomosis vs. Stoma**

**Primary anastomosis is recommended as the first choice in patients with uncomplicated intestinal atresia.**

**Quality of Evidence Range:** Very low

| **Study** | **Type of Study** | **Population** | **Intervention** | **Outcome** | **Key Findings** | **Quality of Evidence** |
| --- | --- | --- | --- | --- | --- | --- |
| Hillyer et al.  J Pediatr Surg.  2019;54:417-422 | Retrospective case series | Neonates w/ jejunal or ileal atresia  mean wt 2.5 kg  (N=92; 60 < 38 weeks GA) | Primary (70) vs secondary (22) anastomosis | 1. Median LOS 2. Median Length of TPN 3. Readmission/Re-op 4. Short (<30 days) and long term (>30 days) complications 5. mortality | 1. 27 vs 95 days 2. 19 vs 74.5 days 3. Lower readmit/re-op rate in PA 4. Overall fewer complications in PA vs SA 5. No difference | Low |
| Del Pin et al. Ann Surg. 1992;215:  179-185. | Retrospective case series | Neonates w/ MI. Mean GA 39 wks. (N=59) | Non-operative (n=11) or surgical (n=48) (ileostomy, resection w/ primary anastomosis) | 1. 6-months survival  2. Long-term survival  3. Effect of selected surgical technique  4. Long-term nutritional assessment between MI vs. non MI CF-pts | 1. Increase since 1960 to 1979-1990 from 33-100%  2. 100% in non-operative cases, in other cases dependent on underlying disease  3. Primary resection anastomosis may have lower surgical morbidity rate  4. MI do not have an adverse effect | Very Low |
| Kimura et al. J Pediatr Surg. 1990;25: 977-979. | Retrospective case series | Neonates w/ duodenal atresia. (N=44) | Diamond shaped anastomosis | 1. Time to postop feeding  2. Survival  3. Complications | 1. Mean 3.7 d  2. & 3. Not related to surgical technique | Very Low |
| Jawaheer et al. Pediatr Surg Int. 2007; 23:1091-1093. | Retrospective case series | Neonates (inc. premature) w/ MI. Mean GA 38 wks. (N=13) | Primary resection and anastomosis | 1. Mean length of time of PN  2. Mean LOS  3. Overall survival  4. Complications | 1. 10.8 d  2. 17.6 d  3. 85%  4. 54% (31% surgical) | Very Low |
| Singh et al. J Pediatr Surg. 2006;41:725-729. | Retrospective case series | Preterm neonates w/ IIP, NEC or undetermined cause of intestinal perforation. Mean GA 28.5 wks. (N=65) | Stoma vs. anastomosis after intestinal perforation | 1. General complications  2. Surgical complications  3. Death | 1. No statistically significant differences | Very Low |
| Hall et al. Arch Surg. 2005;140:1149-1151. | Retrospective case series | Premature infants <1000g w/ NEC. (N=51) | Stoma (n=14) vs. primary resection and anastomosis (n=12) | 1. Short- and long-term survival  2. Length of ICU stay  3. Complications | 1. Comparable outcomes, high mortality in this group of pts | Very Low |

**Antimicrobial Prophylaxis - Impact of antibiotics within 60 minutes**

**Administer appropriate preoperative antibiotic prophylaxis within 60 minutes prior to skin incision.**

**Discontinue postoperative antibiotics within 24 hours of surgery, unless ongoing treatment is required.**

**Quality of Evidence Range:** Low

| **Study** | **Type of Study** | **Population** | **Intervention** | **Outcome** | **Key Findings** | **Quality of Evidence** |
| --- | --- | --- | --- | --- | --- | --- |
| Battin et al. J Paediatr Child Health. 2016;52:913-914. | Cohort, single center | Neonates undergoing abdominal surgery. Median GA 34 wks. (N=60) | Compared abx prophylaxis (n=25) vs. none (n=34) vs. unknown (n=1) | 1. Pre-op abx rate  2. SSI rate | 1. Rate of preop prophylaxis 42%  2. SSI rate 17%  3. SSI w/ abx 8%  4. SSI w/o abx 24% | Low |
| Vu et al. Pediatr Surg Int. 2014;30:587-592. | Cohort, single center | Pts <12 months undergoing thoracic or GI surgery, receiving preop abx prophylaxis (N=732; 40% neonates) | Compared duration of postop abx < 24 h (n=342) vs. >24 hr (n=390) | 1. Overall SSI rate  2. SSI rate based on abx duration | 1. SSI rate 13%  2. No difference in SSI rate based on duration of abx < or >24 hr (9.1% vs. 16.9%) | Low |
| Walker et al. Surgery. 2017;162:1295-1303. | Cohort, single center | Neonates <30 d undergoing GI surgery (N=275) | Implementation of abx prophylaxis protocol  (pre n=148,  post n=127) | 1. Pre-op abx rate  2. Overall SSI rate  3. SSI rate based on abx duration | 1. Rate of preop prophylaxis 99%  2. SSI rate 14%; 9%  3. No difference in SSI rate based on duration of periop abx | Low |
| Segal et al. J Pediatr Surg. 2014;49:381-384. | Cohort, single center | Pts in the NICU undergoing surgery requiring incision.  Median GA 33 wks. (N=1039) | N/A | 1. Overall SSI rate  2. SSI rate by wound class  3. LOS | 1. SSI rate for laparotomies 6.2%  2. Significantly longer LOS w/ SSI vs. no SSI (79 d vs. 25 d, p<0.001) | Low |
| Madden et al. Pediatr Surg Int. 1991; 6:185-189. | Cohort, single center | Neonates <4 wks old undergoing GI surgery (N=143) | Compared rate of SSI in pts not receiving abx vs. those w/ abx | 1. Pre-op abx rate  2. SSI rate  3. SSI rate by wound class | 1. Rate of preop prophylaxis 70%  2. Significantly higher rate of SSI in pts not receiving abx vs. those w/ abx (27.6% vs. 7.6%, p<0.05) | Low |
| Clements et al. J Pediatr Surg. 2016;51:1405-1408. | Cohort, single center | NICU pts undergoing any surgery.  Median GA 33 wks. (N=264) | N/A | 1. Pre-op abx rate  2. SSI rate | 1. Rate of preop prophylaxis 52%  2. SSI rate 11.7%  3. No difference in percent of pts receiving abx in SSI group vs. no SSI | Low |
| Prasad et al. J Perinatol. 2016;36:300-305. | Cohort, 3 centers | Term neonates undergoing GI surgery. Median age at surgery: 5 d. (N=617) | N/A | 1. Pre-op abx rate  2. SSI rate  3. LOS  4. Mortality | 1. Rate of preop prophylaxis 70%  2. SSI rate 5.02%  3. Significantly longer LOS w/ SSI vs. w/o (67.5 d vs. 13 d, p<0.0001)  4. No difference in mortality | Low |
| Davenport et al. J Pediatr Surg. 1993;28:26-30. | Cohort, single center | Term neonates undergoing any surgery from single NICU (N=1433) | N/A | 1. SSI rate  2. SSI rate by wound class | 1. Overall SSI rate 16.6%  2. Significantly higher SSI rate in potentially contaminated wounds as compared to clean wounds (20.9%, 20.5% vs. 11.1%, p<0.001) | Low |
| Alder. ACS Quality and Safety Conference; July 2017. Unpublished. | Cohort, single center. | Neonates undergoing abdo/thoracic surgery (N=62) | Compared pre- (n=35) and post-implementation (n=27) of abx prophylaxis protocol | 1. Pre-op abx rate  2. SSI rate | 1. Rate of preop prophylaxis 49%  2. SSI rate 22.9%  3. Significantly higher rate of SSI in pts not receiving abx vs. receiving abx (39 vs. 6%, p=0.02)  4. No difference in SSI rate or abx prophylaxis pre/post implementation | Low |

**Prevention of Intraoperative Hypothermia**

**Continuously monitor intraoperative core temperature and take pre-emptive measures to prevent hypothermia and maintain normothermia.**

**Quality of Evidence Range:** Very low to low

| **Study** | **Type of Study** | **Population** | **Intervention** | **Outcome** | **Key Findings** | **Quality of Evidence** |
| --- | --- | --- | --- | --- | --- | --- |
| Engorn et al. Pediat Anesth. 2017;27:  196-204. | Prospective intervention study (quality improvement) | NICU surgical pts (N=515)  (excluded cardiac surgical cases) | Transport protocol and OR NICU Transport Temperature Checklist  (pre n=201;  post n=314) | 1. Postoperative hypothermia (First postop temperature recording in the NICU, defined as less than 36°C) | 1. Relative risk reduction of 75% for hypothermia in the post-intervention group  2. PMA and weight were not found to be risk factors for developing hypothermia | Low |
| Morehouse et al. Adv Neonatal Care. 2014;14:154-164. | Prospective case-control study | NICU pts undergoing procedures in the OR (n=55) vs. NICU (n=53) | N/A | 1. Temperatures  2. Frequency of adverse thermo-regulatory, cardiovascular, respiratory, and metabolic outcomes | 1. Pts travelling to the OR are more likely to become hypothermic than those staying in the NICU  2. Hypothermic infants have more respiratory AEs and require more support interventions | Very Low |
| Kim et al. Am J Med Qual. 2013; 28:400-406. | Prospective intervention study (quality improvement) | Pediatric surgical pts (N=7532) | Temperature Management Bundle  (pre n=1758;  post n=2118) | 1. Perioperative hypothermia (core body temp < 36°C on arrival to PACU) | 1. 53% reduction in hypothermia w/ institution of the hypothermia bundle | Low |
| Cassey et al. Pediatr Anesth. 2006;16:  654-662. | Clinical trial | Children undergoing elective surgery under general anesthesia lasting >90 min (N=40) | Modification of the Bair Hugger System w/ a HDU and specific draping technique | 1. Air temperature  2. Skin temperature  3. Core temperature  4. Intraoperative and postop complications | 1. Modified convective heating systems are safe, effective, and low-cost  2. No IV fluid warming, active heating/humidifying of inspired gases or warming blankets were necessary  3. Hyperthermia is a risk | Low |
| Tander et al. Pediatr Anesth. 2005;15:  574-579. | Prospective cohort study | ASA I-III, < 6 months, undergoing pediatric surgical procedures ≥  30 min (N=60) | N/A | 1. Identify risk factors for inadvertent hypothermia | 1. Type of surgery (minor vs. major) and OR temperature (low ‘<23°C’ vs. high ‘>23°C’) are main factors for decreased core temperature in neonates and infants  2. Core temperatures of neonates are less stable than infants, regardless of type of surgery or OR temperature | Very Low |
| Buisson et al. Eur J Appl Physiol. 2004;92:  694-697. | Simulation trial | Manikin  (LBW neonate) undergoing abdominal surgery  (25 trials) | 1. Forced-air warming  2. Warming mattress  3. Surgical sheets | 1. Dry heat loss  2. Local heat loss | 1. Forced-air warming (convection) is more effective than a warming mattress (conduction) (6.8 vs. 2.1 W)  2. Combination of the two resulted in a greater reduction in heat loss (7.9 W) | Very low |

**Perioperative Fluid Management**

**Use perioperative fluid management to maintain tissue perfusion and prevent hypovolemia, fluid overload, hyponatremia, and hyperglycemia.**

**Quality of Evidence Range:** Moderate

| **Study** | **Type of Study** | **Population** | **Intervention** | **Outcome** | **Key Findings** | **Quality of Evidence** |
| --- | --- | --- | --- | --- | --- | --- |
| Larsson et al. Brit J Anaesth. 1990;64:419-424. | RCT | Neonates w/ major congenital defects undergoing surgery in the first wk of life (N=30) | Perioperative fluid regimen of:  1. Ringer-acetate solution (n=15)  2. Ringer-acetate w/ 10% glucose solution (n=15) | 1. Blood glucose  2. Blood gas tensions | 1. Blood glucose concentrations increased in both groups during surgery (group receiving glucose solution increased more)  2. In the ringer-acetate group, intraoperative blood glucose was low if a glucose infusion had been stopped at the start of anesthesia  3. Hypoglycemia was found only in neonates < 48 h of age and only during the first hour of anesthesia  4. Infusion rates were 15-20 ml/kg during the first hour followed by 10 ml/kg/hr in both groups  5. Monitoring blood glucose and adjusting glucose supply seem to be necessary | Moderate |

**Perioperative Analgesia**

**Acetaminophen**

**Unless contraindicated, administer acetaminophen regularly during the early postoperative period (not on an “as needed” basis) to minimize opioid use.**

**Quality of Evidence Range:** Low to high

| **Study** | **Type of Study** | **Population** | **Intervention** | **Outcome** | **Key Findings** | **Quality of Evidence** |
| --- | --- | --- | --- | --- | --- | --- |
| Ceelie et al. JAMA. 2013;309:149-154. | RCT (double-blind, single-center) | Neonates or infants post  major thoracic (non-cardiac) or abdominal surgery (N=71; 77% abdominal procedures) | 1. Loading dose morphine and continuous morphine (n=38)  2. Loading dose morphine and  intermittent IV paracetamol (n=33) | 1. Cumulative morphine dose  2. Pain scores  3. AE | 1. Significantly reduced cumulative morphine dose in paracetamol group vs. morphine group (121 vs. 357 µg/kg, p<0.001).  2. Between group difference of 66% (CI 34-109%)  3. No significant difference in pain scores and AE between groups | High |
| Allegaert et al. Pediatr Anesth. 2013;23:45-50. | Prospective, single-center open label study | Neonates (<29 d) receiving paracetamol as analgesic  (N=60) | 1. Paracetamol  loading (20mg/kg) and  maintenance (20-40mg/kg/d in 4 divided doses) | 1. Pain scores | 1. IV paracetamol effective for moderate pain  2. Similar effect compartment concentration in neonates as for children (10mg/L) | Low |
| Allegaert et al. Arch Dis Child. 2011;96:575-580. | Pharmaco-kinetic study  (4 studies) | Neonates (27-45 PMA) (N=158) | 1. Single dose IV propacetamol on day 1 (n=30)  2. Repeated IV propacetamol (n=18)  3. Repeated IV paracetamol (n=50)  4. Repeated IV paracetamol (n=60) | 1. Pharmaco-kinetic profile | 1. Patient size as predicted by weight is the major covariate of clearance variance in neonates  2. An increased volume of distribution supports the use of a loading dose  3. A mean paracetamol concentration of 11mg/l is predicted after 10mg/kg IV every 6 hours (neonates 32-44w PMA) | Low |

**Opioids**

**Use an opioid-limiting strategy is recommended in the postoperative period. Manage breakthrough pain with the lowest effective dose of opioid with continuous monitoring.**

**Quality of Evidence Range:** Very low to moderate

| **Study** | **Type of Study** | **Population** | **Intervention** | **Outcome** | **Key Findings** | **Quality of Evidence** |
| --- | --- | --- | --- | --- | --- | --- |
| Lynn et al. Pain. 2000; 88:89-95. | RCT | Surgical infants (N=83) | 1. Intermittent IV morphine (0.05mg/kg) (n=27)  2. Continuous IV morphine (20ng/ml) (n=56) | 1. Modified infant pain scores  2. HR, SpO2  4. M6G  5. Time to oral intake  6. Pruritus  7. Side effects | 1. Both groups achieved pain scores consistent w/ analgesia but bolus group showed higher % of pain scores indicating distress (32 vs. 13%, p<0.001)  2. Large interindividual variability in morphine plasma levels (clearance increased w/ age)  3. Most infants had no notable side effects | Moderate |
| Bouwmeester et al. Intensive Care Med. 2003;29:  2009-2015. | RCT  double-bind,  single center | Full term neonates post major surgery (N=68)  [22/68 (32%) underwent intestinal resection procedures] | 1. Continuous morphine  (10µg/kg/h)  2. Intermittent morphine (30µg/kg/3h) | 1. Pain scores (Comfort behavioral scale/ VAS)  2. Morphine/ M6G plasma concentrations | 1. Younger neonates (≤7 d) differed significantly from the older neonates in median morphine requirement (10.0 vs. 10.8 µg/kg/h), morphine plasma concentration (23.0 vs. 15.3 ng/ml) and M6G/morphine ratio (0.6 vs. 1.5)  2. No difference in pain scores between age groups or morphine treatment groups  3. Both morphine regimes equally effective and safe | Moderate |
| Krekels et al. Clin Pharmacokinet.  2014;53:553-563. | Pharmaco-kinetic study | Term neonates and infants undergoing major abdominal/ non-cardiac thoracic surgery (N=38) | Model-derived morphine dosing algorithm | 1. Pain score  2. Morphine rescue | 1. Efficacy: 5/18 (27.8%) neonates w/ PNA <10 d needed rescue medication vs. 18/20 (90%) of older pts  2. Median total morphine rescue dose was 0 (range 0-20) µg/kg vs. 193 (19-362) µg/kg | Moderate |
| Morton et al. Paediatr Anaesth. 2010; 20:119-125. | Prospective audit; UK centers | Children (0-18 y) receiving PCA, NCA (N=10,726)  Neonates (<1 m) (n=688; 6.4%) | Postoperative opioid infusion techniques (PCA, NCA) | 1. Incidence/ severity of serious clinical incidents | 1. Overall incidence of serious harm 1:10,000  2. 46 serious clinical incidents (neonates: cardiac arrest n=1, respiratory depression n=1) | Very low |
| Howard et al. Paediatr Anaesth. 2010; 20:126 | Prospective review;  single center | Children (1d-20y) receiving morphine NCA post major surgery (N=10,079) | Morphine NCA | 1. Effectiveness  2. Morphine requirements  3. Incidence of side effects/AE | 1. SAE 0.4%  2. SAE significantly greater in neonates (2.5%), (RR=9.4; p<0.001)  3. No significant difference in morphine dose in neonates who experienced SAE vs. those who did not | Very low |
| Saarenmaa et al. J Pediatr. 1999;134:  144-150. | RCT;  double-blind | Newborn infants (N=163) | 1. Fentanyl infusion (10.5µg/kg loading then 1.5µg/kg/h) (F)  2. Morphine (140µg/kg then 20µg/kg/h) for ≧ 24h (M) | 1. Pain severity (physiological parameters, behavioural pain scale)  2. Stress hormone concentrations at 0, 2, 24 h  3. GIT motility | 1. Analgesic effect similar in both groups  2. Median adrenaline decrease from 0 to 24 h was 0.5nmol/L (F) and 0.7nmol/L (M); Median noradrenaline decrease was 2.1nmol/L (F) and 3.0nmol/L (M)  3. Decreased GIT motility less frequent in fentanyl group (23% vs. 47%, p<0.01) | Moderate |

**Standard Anesthetic Protocol**

**Use regional anesthesia and acetaminophen perioperatively in combination with general anesthesia. Multi-modal strategies including regional techniques should be continued postoperatively.**

**Quality of Evidence Range:** Very low to high

| **Study** | **Type of Study** | **Population** | **Intervention** | **Outcome** | **Key Findings** | **Quality of Evidence** |
| --- | --- | --- | --- | --- | --- | --- |
| Somri et al.  Pediatr Anesth. 2007;17:1059-1065. | Clinical single arm trial | Premature and full-term neonates and infants (ASA I-III) undergoing elective primary abdominal surgery (N=28) | Combined spinal-epidural anesthesia (spinal anesthesia w/ isobaric bupivacaine 0.5%, 1mg/kg + caudal epidural catheter to thoracic spinal segments w/ plain bupivacaine) | 1. Cardio-respiratory outcomes  2. Postoperative opioid use  3. Conversion to general anesthesia  4. Time to motor and sensory block  5. Midazolam use | 1.Surgical anesthesia was achieved in 24/28 pts (4 were converted to general anesthesia)  2. Combined spinal-epidural anesthesia leads to decreased opioid use  3. 20/24 pts having regional anesthesia required IV midazolam; pts required oxygen supplementation and transient manual ventilation intraoperatively | Low |
| Somri et al.  Pediatr Surg Int. 2011;27:1173-1178. | RCT | NICU  pts undergoing elective primary GI surgery (N=50) | General anesthesia (n=25) vs. Combined spinal-epidural (n=25) | 1. Postoperative cardiorespiratory AE | 1. More total postop respiratory AE and more infants who experienced ≥1 respiratory AE in general anesthesia group (p<0.0001)  2. More cardiovascular AE in general anesthesia group (p=0.005) | Moderate |
| Somri et al.  J Clin Anesth. 2012;24:439-  445. | RCT | Young infants undergoing elective intestinal surgery (N=50) | General anesthesia  (n=25) vs.  Combined spinal-epidural (n=25) | 1. Time to first postop stool  2. Duration of NG feeding  3. Time to full enteral feeds  4. Postop AE  5. Postop opioid requirements | 1. Recovery of intestinal function was faster (p<0.0001) and the frequencies of postop abdominal distension and pneumonia were less (p<0.04) in pts w/ CSE  2. Continuous fentanyl infusion was required in 100% of GA (median: 250 μg) vs 12% of CSEA (median: 60 μg) patients | Moderate |
| Calder et al.  Pediatr Anesth. 2011;22:430-  437. | RCT/ Pharmaco-kinetic study | PMA <66 wks undergoing elective hernia repair or abdominal surgery under general anesthetic w/ epidural blockade (N=31) | Single epidural shot of 1.5 mg/kg 0.25% bupivacaine vs. 0.2% ropivacaine  (+ 0.2 mg/kg/hr infusion 2 hrs later for abdominal cases) | 1. Pharmaco-  kinetic profile  2. Pain scores  3. AE | 1. Epidural infusions of both local anesthetics were well tolerated and efficacious  2. No accumulation of unbound drug concentrations occurred | Moderate |
| Maitra et al. Journal of Anesthesia. 2014;28:768-  779. | Systematic review  (16 studies) | Surgical term and preterm neonates | Epidural anesthesia and analgesia | 1. Satisfactory surgical condition  2. Ventilation requirements  3. Intraoperative and postop complications | 1. Continuous epidural anesthesia for major thoracic and abdominal surgery offers good postop analgesia, as well as early extubation, attenuation of the stress response, early return of bowel function  2. Risk of procedure-related and drug-related complications are a serious concern | Moderate |
| Polaner et al. Anesth Analg. 2012;115:1353-1364. | Prospective cohort | Children receiving regional anesthetic by an anesthesiologist (N=13725) | Intraoperative therapeutic and diagnostic nerve blocks, postop continuous blocks | 1. Intraoperative and postop complications | 1. There were no deaths or complications w/ sequelae lasting >3 months. | Low |
| Fredrickson et al. Anaesth  Intensive Care. 2009;37:469-  472. | Case series | Neonates undergoing abdominal surgery w/ a TAP block (N=4) | Intraoperative and early postop analgesia via TAP in conjunction w/ general anesthesia | 1. HR, MAP, ETMAC, day of extubation, POD0 feed  2. Intra- and post-operative analgesia | 1. TAP blocks provided effective analgesia in all 4 pts w/ only 1 requiring perioperative opioids (large gastroschisis w/ elective postop ventilation) | Very low |
| Jacobs et al.  Pediatr Anesth. 2011;21:1078-1080. | Case series | Neonates and infants undergoing abdominal surgery w/ a TAP block (N=10) | Ultrasound-guided TAP block w/ 1ml/kg of 0.25% levobupivacaine | 1. Intraoperative opioid requirement  2. Postoperative pain scores (NIPS)  3. Total analgesic requirement for the first 24 hrs postop | 1. 7/10 pts did not require intraoperative opioids  2. Procedures involving extensive bowel handling require opioids for visceral pain; opioid requirements may be reduced by preop TAP block | Very low |
| Allegaert et al. Arch Dis Child. 2011;96:575-  580. | Pharmaco-kinetic studies  (4 studies) | Extreme preterm, preterm, and term neonates prescribed propacetamol or paracetamol (N=158) | 1. Single dose IV propacetamol on day 1 (n=30)  2. Repeated IV propacetamol (n=18)  3. Repeated IV paracetamol (n=50)  4. Repeated IV paracetamol (n=60) | 1. Pharmaco-kinetic profile  2. Safety | 1. An increased volume of distribution supports use of a loading dose in neonates  2. Size is the major covariate contributing to paracetamol clearance variance in neonates  3. Authors suggest a loading dose of 20 mg/kg followed by 10 mg/kg every 6 hours in neonates 32-44 wks’ PMA  4. Safety data is limited | Low |
| Ceelie et al.  JAMA. 2013;309:149-  154. | RCT | Neonates or infants <1 yr undergoing major thoracic (noncardiac) or abdominal surgery (N=71) | 1. Loading dose of morphine and continuous morphine (n=38)  2. Loading dose of morphine and intermittent IV paracetamol (n=33) | 1. Cumulative morphine dose  2. Pain scores  3. Morphine-related adverse effects | 1. Cumulative median morphine dose within 48 hours postop was 121 mcg/kg in the paracetamol group and 357 mcg/kg in the morphine group (p<0.001)  2. No significant differences in pain scores and adverse effects | High |
| Zhu et al. Anesth Analg. 2017;125: 1569-1587. | Systematic review  (35 studies) | Pediatric surgical pts undergoing general anesthesia | Pre or intraoperative single, systemic, non-opioid medication vs. opioid vs. placebo | 1. Postop narcotic requirements or pain scores  2. Time to first analgesic use  3. Postop nausea | 1. Data support the use of acetaminophen, NSAIDs, dexamethasone, ketamine, clonidine, and dexmedetomidine to decrease postop pain and/or opioid consumption (n=11 studies for acetaminophen)  2. Of these, only acetaminophen has been studied in neonates | High |

**Lingual Sucrose/Dextrose**

**Provide lingual sucrose/dextrose to reduce pain during naso/orogastric tube placement and other minor painful procedures.Quality of Evidence Range:** Low to high

| **Study** | **Type of Study** | **Population** | **Intervention** | **Outcome** | **Key Findings** | **Quality of Evidence** |
| --- | --- | --- | --- | --- | --- | --- |
| Harrison et al. Pediatrics.  2017; 139:  e20160955. | Meta-analysis  (168 studies) | Term and/or preterm infants (N=1175 for crying time);  (N=3341 for pain scores) | Infants randomized to treatment groups (sucrose of glucose) or control groups | 1. Cry duration  2. Pain scores | 1. Mean differences in cry time was -23.18s in favor of sweet solutions (95% CI -28.89 to  -17.47)  2. SMD of -0.90 in favor of sweet solutions over control or placebo (95% CI -1.09 to -0.70) | High |
| Stevens et al. Cochrane Database  Syst Rev. 2016;7: CD001069. | Systematic review  (74 studies) | Term or preterm neonates (N=7049) | Sucrose for procedural pain. Controls included no treatment, water, glucose, breast milk, breastfeeding, local anesthetic, pacifier, positioning, or acupuncture. | 1. PIPP  2. AE | 1. Beneficial effect of 24% sucrose w/ non-nutritive sucking or 0.5 mL of sucrose orally (PIPP 30s after heel lance WMD -1.70; PIPP 60s after heel lance WMD -2.14).  2. Beneficial effect of 2 mL 24% sucrose prior to venipuncture (PIPP during venipuncture WMD -2.79, PIPP during intramuscular injection WMD -1.05)  2. AE were minor and similar in the sucrose and control groups | Moderate |
| McCullough et al. Arch Dis Child Fetal Neonatal Ed. 2008;93:  F100-103. | RCT  (double-blind, placebo controlled clinical trial\|) | Stable preterm infants who required NGT insertion for feeding  (N=20) | Randomized to lingual 24% sucrose vs. water administered 2 mins before NGT insertion | 1. HR  2. SaO2  3. NFCS  4. Presence or absence of crying  5. Adverse effects | 1. Sucrose-treated infants have little change in mean HR vs. placebo (−0.73 vs.11; p = 0.055)  2. No significant change in SaO2  3. Sucrose-treated infants had lower NFCS vs. placebo (p = 0.004)  4. Non-significant trend (p = 0.069) for fewer sucrose-treated infants to cry w/ NGT insertion vs. placebo  5. AE were few and occurred equally in each group | Moderate |
| Ravishankar et al. J Paediatr Child Health. 2014;50:  141-145. | RCT (double‐blinded, placebo‐controlled) | Neonates (N=150) | NGT insertion after giving 2 mL of:  1. 25% dextrose (D25)  2. 10% dextrose (D10)  3. Placebo (distilled water) | 1. PIPP score  2. Cry duration  3. Change in HR  4. SpO2 | 1. D25 had lesser pain response to NGT insertion in terms of lower PIPP score (p<0.05) and duration of cry (p=0.001) compared to D10. Also smaller increase in HR and decrease in SpO2 (p<0.05).  2. In comparison w/ placebo, D10 decreased duration of cry (p<0.05) but not PIPP score. | High |
| Gibbins et al. Nursing Research. 2002;51:  375-382. | RCT | Neonates (N=190) | 1. Sucrose and nonnutritive sucking (n=64)  2. Sucrose alone (n=62)  3. Sterile water and nonnutritive sucking (control) (n=64) | 1. PIPP  2. AE | 1. Significant differences in pain response existed among treatment groups (F=22.49, p<0.001), w/ the lowest mean PIPP scores in the sucrose and nonnutritive sucking group following heel lance. | Moderate |
| Carbajal et al. BMJ.  1999;319:  1393-1397. | Randomized prospective study | Term newborns undergoing venipuncture (N=150) | Six treatment groups:  1. No treatment  2. Placebo (2 ml sterile water)  3. 2 ml 30% glucose  4. 2 ml 30% sucrose  5. Pacifier  6. 2 ml 30% sucrose followed by pacifier | 1. Pain scores (DAN Scale) | 1. Median pain scores during venipuncture were 7 (range 5-10) for no treatment; 7 (6-10) for placebo (sterile water); 5 (3-7) for 30% glucose; 5 (2-8) for 30% sucrose; 2 (1-4) for pacifier; and 1 (1-2) for 30% sucrose plus pacifier. | Low |
| Stevens et al.  Clin J Pain. 2005;21:  543- 548. | RCT | Preterm infants (N=66) | Prior to painful procedures:  1. Standard care (positioning and swaddling; n=21)  2. Sterile water plus pacifier (n=23)  3. 24% sucrose plus pacifier (n=22) | 1. PIP  2. AE | 1. Significant main effect of intervention (p=0.03) between the sucrose plus pacifier group, and the standard care group (p=0.01), but there was no main effect of time (p=0.72).  2. No group differences existed for AE, clinical outcomes, or neurobiological risk status. | Moderate |
| Johnston et al. Pediatrics. 2002;110:  523- 528. | RCT | Preterm neonates <31 wks (N=107) | Oral sucrose (24%) or sterile water administration up to 3 times, 2 min apart, for every invasive procedure | 1. Neuro-behavioral assessment  2. Score for Neonatal Acute Physiology  3. NBRS | 1. No significant differences | Moderate |
| Işik et al.  J Pain. 2000;  1:275-278. | RCT | Healthy term newborns (N=113) | 4 groups - 2 ml of:  1. 30% sucrose  2. 10% glucose  3. 30% glucose  4. Distilled water | 1. Mean crying time | 1. Mean crying times were 60, 102, 95, and 105 seconds in the groups 1, 2, 3, and 4 respectively (p=0.02) | Moderate |
| Taddio et al. CMAJ. 2008;179:  37-43 | RCT | Newborns ≥  36 wks gestation (N=240) | 2 mL of 24%-sucrose or placebo solution before all procedures | 1. PIPP | 1. Overall mean pain score was lower in sucrose group vs. placebo (MD -1.3, 95% CI -2.0 to -0.6) | High |

**Optimal Hemoglobin**

**Restrict transfusions to maintaining HgB >/=90 (9 g/dL for a term neonate with no oxygen requirement. Term neonates within the first week of life, intubated or with an oxygen requirement should be transfused to maintain a HgB >/=110 (11 g/dL).**

**Use written transfusion guidelines and take into account not only a target hemoglobin threshold, but also the clinical status of the neonate and local practices.**

**Quality of Evidence Range:** Low

| **Study** | **Type of Study** | **Population** | **Intervention** | **Outcome** | **Key Findings** | **Quality of Evidence** |
| --- | --- | --- | --- | --- | --- | --- |
| Bell et al. Pediatrics. 2005;115: 1685-1691. | RCT | Preterm infants 500 to 1300 g (N=100) | Restrictive (low; n=49) vs. liberal (high; n=51) Hct thresholds for RBC transfusions | 1. RBC transfusions  2. Number of donors  3. Hemoglobin  4. Hematocrit | 1. RBC transfusions less in  restrictive-transfusion group  2. Number of donors exposure not significantly different  3. Hgb, g/dL, mean & SD 11.0 +/-1.9 vs 8.3+/- 1.1  4. Hct, %, mean & SD 32 +/- 6 vs 26 +/- 5 | Low |
| McCoy et al. Child Neuropsych. 2011;17: 347-367. | RCT | Preterm infants  (N=56) | Restrictive (low; n=33) vs. liberal (high; n=23) Hct thresholds for RBC transfusions | 1. Neurocognitive profiles | 1. RBC transfusions affected the long-term outcome of premature infants as indicated by reduced brain volumes at 12 y old for neonates who received transfusions w/ liberal guidelines | Low |
| Kirpalani et al. J Pediatrics. 2006;149: 301-307.e3. | RCT | Extremely LBW preterm infants (<1000g) and <31 wks GA (N=451) | Restrictive (low; n=223) vs. liberal (high; n=228) Hgb thresholds for RBC transfusions; Transfused following an algorithm where the highest Hgb threshold was 13.5g/l or Hct 40.5%, and the lowest Hgb threshold was 7.7g/l or Hct 23% | 1. Hemoglobin  2. Morbidity/ Mortality  3. RBC transfusions | 1. Mean Hgb difference before transfusion was 1.1 g/dL  2. Short-term outcomes showed no difference in death or major morbidity with restrictive or liberal transfusion regimens.  3. Fewer infants (89%) in the low threshold group had received a transfusion, compared with the high threshold group (95%) | Low |
| Whyte et al. Pediatrics. 2009;123: 207-213. | RCT | Extremely LBW infants (N=421) | Restrictive (low; n=208) versus liberal (high; n=213) Hgb thresholds for RBC transfusions | 1. Primary Outcome: death, cerebral palsy, cognitive delay, severe visual or hearing impairment | 1. No significant difference in primary outcome; found in 45% in the restrictive group and 38% in the liberal group.  2. Post-hoc analysis with cognitive delay redefined (Mental Development Index Score < 85) showed a significant difference favoring the liberal threshold group | Low |
| Goobie et al. JAMA Pediatrics. 2016;170: 855-862. | Database analysis | Neonates w/ a preop Hct in NISQIP database  (N=2763) | Compared anemic neonates (Hct <40%) vs. nonanemic neonates | 1. In-hospital mortality | 1. Preoperative anemia is independently associated with postoperative in-hospital mortality (OR=2.6; 95%CI 1.5-4.6). | Low |

**Perioperative Communication and Team Structure**

**Implement perioperative multidisciplinary team communication with a structured process and protocol (“pre- and post-operative huddle”) utilizing established checklists.**

**Quality of Evidence Range:** Low to moderate

| **Study** | **Type of Study** | **Population** | **Intervention** | **Outcome** | **Key Findings** | **Quality of Evidence** |
| --- | --- | --- | --- | --- | --- | --- |
| Singer et al. J Am Coll Surg. 2016;223:568-580. | Prospective cohort | Hospital staff in 10 hospitals; 207 procedures (Safe Surgery South Carolina Initiative)  (N=207) | 1. SSC Implementation: webinar, meeting, training, coaching  2. Measured and monitored baseline and follow-up safe surgical practices | 1. Checklist completion rates and association w/ surgeon buy-in and teamwork  2. Safe surgical environment | 1. Surgeon engagement and consistent team work most important for promoting checklist use and ensuring safe surgical environment | Moderate |
| Ventre et al. Simul Healthc. 2014;9:102-111. | Prospective intervention study | 133 HCW from a single site children's hospital in Colorado  (N=133) | 3 simulation scenarios; evaluated  pre- and post-intervention | 1. Operational readiness | 1. Multiple operational deficiencies identified  2. Decreased time to respond to emergent response (time between transfusion protocol activation and blood arrival) | Low |
| Mancuso et al. J Obstet Gynecol Neonatal Nurs. 2016;45:502-514. | Prospective intervention study | Obstetric and neonatal staff attending cesarean deliveries (N=102;  pre n=52,  post n=50) | Team training in crew resource management, critical language, communication, and team structure | 1. Quantity and quality of communication during cesarean deliveries | 1. Improvement in quantity and quality of communication.  2. Increase in quality was similar between obstetric and neonatal staff, but increase in quantity was greater in the obstetric staff | Low |
| Brodsky et al. BMJ Qual Saf. 2013;22:374-382. | Prospective intervention study | NICU staff in Boston  (N=218;  pre n=114;  post n=104) | Team work training workshop | 1. Pre and post workshop staff survey | 1. Improved communication, awareness of unit acuity, improved overall teamwork score | Low |
| Segall et al. Anesth Analg. 2012;115:102-115. | Systematic review of mostly comparative studies w/o historical controls  (14 studies) | Postop handovers  (4 studies w/ interventions shown below: Catchpole, Joy, Mistry, Zavalkoff) | N/A | 1. Review of recommendations of postop handovers | Broad support for:  1. Standardize processes (use of checklists and protocols)  2. Complete urgent clinical tasks before info transfer  3. Allow only patient-specific discussions during handover  4. Require all relevant team members be present  5. Provide training in team skills and communication | Moderate |
| Catchpole et al. Pediatr Anesth. 2007;17:470-478. | Prospective intervention study | 50 postop handovers to cardiac ICU (pre n=23;  post n=27) | New handover protocol utilizing Formula 1 racing team and aviation training captains | 1. Technical errors  2. Info omissions  3. Teamwork  4. Duration of handover | 1. Reduction in technical errors, info omissions, and duration of handover | Low |
| Joy et al. Pediatr Crit Care Med. 2011;12:304-308. | Prospective  intervention study | 79 postop handovers from OR to CICU  (pre n=41;  post n=38) | Teamwork driven handover process and protocol | 1. Technical errors  2. Info omissions  3. Duration of handoff  4. Caregiver rating of teamwork | 1. Significant reductions in technical errors and handoff info omissions.  2. No change in duration of the handover process.  3. Caregivers noted improvement in teamwork | Low |
| Mistry et al. Adv Pat Saf 2008;3. | Prospective  intervention study | 141 postop handovers  (pre n=29;  post n=142) | Improved handoff process and medical simulation training (refer to abstract from Critical Care Congress 2006) | 1. Duration of handoff  2. Time to obtain critical lab studies  3. % pts placed on cardioresp monitoring | 1. Improved handoff and decrease in the delay of time-sensitive therapies | Low |
| Zavalkoff et al. Pediatr Crit Care Med. 2011;12:309-313. | Prospective interventional study | 33 HCP participating in 31 handovers | One page tool to guide info transmitted by surgeon and anesthesiologist to PICU team; postop cardiac pts | 1. Total handover score  2. Handover duration  3. Postop high-risk events | 1. Significant improvement in handover score, and medical and surgical intraoperative info subscores  2. No change in duration  3. Trend for decrease of high-risk events | Low |
| Petrovic et al. J Cardiothor Vasc Anesth. 2012;26:11-16. | Prospective interventional study | 238 HCP during the transfer of 60 pts  (pre n=169;  post n=137) | Standardized handoff protocol and checklist | 1. Staff presence  2. Missed info  3. Satisfaction  4. Duration of procedure | 1. Increased staff presence  2. Decreased missed info (surgery, not anesthesia)  3. Increased satisfaction  4. Increased duration of handoff | Low |
| Agarwal et al. Crit Care Med. 2012;40:2109-2115. | Prospective interventional study | Postop pediatric cardiac surgery; responses  (pre n=61;  post n=114) | Pre-intervention: verbal handover  Post-intervention: structured handover | 1. Info transfer  2. Quality of handover (Likert)  3. Patient complications within first 24 hours | 1. Quality of handover excellent  2. Reduction in loss of info  3. Decrease in major complications  4. Increase early extubations | Low |

**Parental Involvement**

**Facilitate hands on care and purposeful practice by parents that is individualized to meet the unique needs of parents early during the admission. Sustain these to build the knowledge and skills of parents to take on a leading role as caregivers and facilitate their readiness for discharge.**

**Quality of Evidence Range:** Very low to high

| **Study** | **Type of Study** | **Population** | **Intervention** | **Outcome** | **Key Findings** | **Quality of Evidence** |
| --- | --- | --- | --- | --- | --- | --- |
| Glazebrook et al. Arch Dis Child Fetal Neonatal Ed. 2007;92:F438  -F443. | Cluster RCT w/ crossover and a 3-mo washout period | Parents to infants born at <32 wks from 6 NICU. (intervention phase n=112; control phases n=121) | Parent Baby Interaction Program; educational intervention and optional home follow-up 6 wks | 1. Parenting stress at 3 months  2. Neurobehavioral assessment of preterm infant and maternal interaction and responsivity subscale | 1. No difference in any of the outcomes. Authors questioned whether the dose of intervention too low or timing was too early (do just before or after discharge) | High |
| Pfander et al. Children's health care: journal of the association for the care of children's health. 1990; 19:140-146. | Prospective cohort | NICU pts and their parents, Central Michigan  (N=48) | Four groups:  1. Parent education and infant assessment  2. Parent education  3. Infant assessment  4. Control | 1. Bayley Mental Scale at 6 months  2. Bring for regular check-ups  3. Nutrition | 1. Parent education was associated w/ increased scores on the Bayley Mental Scale  2. Parents in the treatment groups provided better nutrition to their infants | Moderate |
| Ingram et al. BMJ Open. 2016;6:  e010752. | Prospective intervention study | 245 families of premature neonates in 4 NICUs  (pre n=128;  post n= 117) | Family-centered discharge teaching package implemented | 1. PMPS-E score  2. LOS  3. Healthcare utilization post discharge | 1. Decreased ED visits in post phase families  2. No change in other measures | Low |
| Raines et al. MCN*.* 2017; 42:95-100. | Prospective intervention study | Mother/father dyads about to be discharged from the NICU (N=15) | Simulation experience | 1. Confidence scores and evaluation | 1. Simulation made the parents feel more prepared  2. Fostered a positive transition to home | Low |
| Franck et al. J Perinat Neonatal Nurs. 2017; 31:244-255. | Descriptive qualitative | Parents from 7 NICUs; Northern Ireland (N=40) | Parent focus groups using protocolized approach to describe experiences | 1. Parental experiences | 1. Discharge from NICU was described as sudden and rushed  2. Inconsistency in communication noted  3. Need for further help w/ follow up and post discharge issues identified | Very low |
| Franck et al. Pediatrics. 2011;128:510. | Descriptive qualitative (RCT) | Parents from 4 NICUs;  control (n=85) and intervention (n=84) | Parents in intervention group received a booklet on managing pain and 2 visits by research RN | 1. Parent questionnaires, including PSS:NICU | 1. No difference in stress, but significant difference in role attainment post discharge and increase in parent satisfaction | High |
| Browne et al. J Pediatr Psychol. 2005;30:667-677. | RCT | Mother-baby dyads from NICU randomized to one of 2 interventions or control. (N=84) | 1. Demo and interaction  2. Educational materials  3. Control | 1. Parent questionnaires including KPIB, PSI, and NCAFS | 1. No difference in PSI but elevated across all groups  2. Both intervention groups did better w/ knowledge (KPIB) and had better interactions w/ infant (NCAFS) | High |
| Abdel-Latif et al. Arch Dis Child Fetal Neonatal Ed. 2015;100:  F203-F209. | Cross over RCT and focus group | Parents of infants in ICU w/ anticipated stay >11 d; 39 HCP  Focus group: 9 HCP, 8 parents  (N=72) | Parent presence at bedside rounds vs. not | 1. PSS:NICU  2. Satisfaction survey | 1. Parents and HCP strongly support parental presence  2. Should develop policies to address decreased educational opportunities and confidentiality concerns | High |
| Raiskila et al.  Early Hum Dev. 2014;90:  863-867. | Retrospective cohort | Preterm infants in 4 cohorts:  2001-2 (n=72) 2006-7 (n=69) 2009-10 (n=76) 2011-12 (n-78) | N/A | 1. Trends in FCC practices | 1. Decreased GA at end of incubator care and start of BF, decreased bottle feeding, increased STS care, increased weight gain | Low |
| Voos et al. J Matern Fetal Neonatal  Med. 2011;  24:1403-1406. | Comparative w/o concurrent controls | Parents and staff from NICU in Kansas City  Staff: (pre n=142, post n=136)  Parents: (pre n=12, post n=16) | Implementation of family-centered rounds | 1. Provider satisfaction and collaboration  2. Parent satisfaction w/ provider communication | 1. Enhanced collaboration among teams  2. Increased parent satisfaction for communication | Low |
| Penticuff et al. J Perinat Neonatal Nurs. 2005;19:187-202. | Comparative: quasi-experimental repeated measures design, prospective | Triethnic sample of mothers of 154 LBW babies (<=1500g) (intervention n=77;  control n=77) | Infant progress chart and core planning meetings between parents and providers | 1. Comprehension of infant medical condition  2. Satisfaction w/ collaboration in treatment decisions | 1. Improved collaboration assessment and accuracy of parent understanding  2. Intervention most effective in low income, young minority mothers | Moderate |
| Weis et al. Nursing in Critical Care. 2015;20:287-  298. | Qualitative interview and prospective cohort | Parents of premature infants 10 dyads (n=20)  (intervention n=13; control n=9) | GFCC vs. standard care | 1. Parent experiences via interviews | 1. GFCC helped parents discover and express emotions, reach a deeper level of communication, and obtain mutual understanding | Moderate |
| Schweitzer et al. J Pediatr Health Care. 2014;28:420-  428. | Prospective  intervention study | Pediatric pts  (0-17 y) w/  G-tubes (n=26)  Historical control (n=23) | Pre-procedure education protocol | 1. Patient outcomes  2. Caregiver knowledge and confidence  3. Provider satisfaction | 1. Improved patient outcomes, caregiver knowledge, and confidence | Low |
| Weiss et al. J Perinatol. 2010;30:425-  430. | Prospective  intervention study | Parents in NICU, San Diego CA;  (pre n=50,  post n=33) | Intervention to improve patient satisfaction w/ provider communication | 1. Parent satisfaction w/ primary medical provider communication | 1. More subjects in the post-intervention cohort were satisfied (95%) w/ provider communication than in the pre-intervention cohort (74%; p<0.01) | Low |
| Globus et al. J Perinatol. 2016;36:739-  743. | Prospective  intervention study | Parents in NICU, Israel  (pre n=91,  post n=87) | Daily SMS texts to update parents about their preterm infant | 1. Parent questionnaires regarding satisfaction w/ communication | 1. Parents felt the physician was more available and approachable  2. Overall more satisfied w/ medical info | Low |
| Aagaard et al. J Pediatr Nurs. 2008;23:e26-  e36. | Meta-synthesis of qualitative studies | 14 studies of mothers’ experiences of having a preterm infant | N/A | 1. Experiences of mothers | 1. Reciprocal relationships for mother baby, maternal development, turbulent neonatal environment, maternal caregiving and role reclaiming strategies and mother-nurse relationship | Low |
| Provenzi et al.  J Clin Nurs.  2015;24:1784-  1794. | Systematic review of qualitative studies | 14 studies of fathers’ experiences of having a preterm infant in NICU | N/A | 1. Experiences of fathers | 1. Fathers experience ambivalence, different needs and coping strategies  2. Need targeted nursing support and interventions to sustain caregiving engagement and transition to parenthood | Low |
| Sisson et al. J  Obstet Gynecol Neonatal Nurs. 2015; 44:471-480. | Meta-ethnographic synthesis; Primary qualitative studies (24 studies) | Studies of fathers’ experiences of having preterm infants in NICU | N/A | 1. Experiences of fathers | 1. Fathers have a need to interact and be involved; Staff in NICU can play key role in facilitating this through encouragement and reassurance | Low |
| Mouradian et al. Am J Occup Ther. 2013;67:  692-700. | Prospective intervention study; Qualitative descriptive | Parents of premature neonates in level 3 NICU in US (N=40) | Art therapy for parents | 1. Pre- and post- activity STAI  2. Interviews post- activity | 1. Significant reduction in STAI score post-activity  2. Parents reported that activity provided distraction and reduced isolation | Low |
| Ardal et al.  Neonatal Netw. 2011; 30:89-98. | Qualitative descriptive | Non anglophone mothers (N=8) | Same language peer support buddy | 1. Parent questionnaire | 1. Language barriers compound difficulties w/ guilt, anxiety, linguist | Very low |
| Kerr et al. BMC Pediatr. 2017;17:158-13. | Prospective qualitative | Parents of preterm neonates in Scotland (N=133) | Introduction of webcams in NICU | 1. Interviews w/ families | Most families found it helpful, a few families did not | Very low |

**Postoperative Nutritional Care**

**Early Feeding**

**Start early enteral feeds within 24-48 hours after surgery when possible. Do not wait for formal return of bowel function**

**Quality of Evidence Range:** Very low to high

| **Study** | **Type of Study** | **Population** | **Intervention** | **Outcome** | **Key Findings** | **Quality of Evidence** |
| --- | --- | --- | --- | --- | --- | --- |
| Ekingen, G et al. Nutrition. 2005;21:142-146. | RCT | Newborns undergoing upper abdominal operations  (N=56) | 1. EEN (3-5 mL of breastmilk/hr via NGT starting 8-20 hr after surgery (n=33)  2. Traditional Protocol (n=23) | 1. Daily gastric drainage  2. Time to first stool  3. Day of toleration of full oral feeding  4. LOS | 1. Shorter time to first stool (31.9 vs. 55.3h) and LOS (10.8 vs. 17.6d) in the EEN groups  2. Intestinal anastomosis group showed significantly shorter duration of NG feeding (6.9 vs. 13.6d) and time to full oral feeds (8.4 vs. 15.4d) in EEN group | High |
| Dunn et al. J Pediatr. 1988;112: 622-629. | RCT | VLBW neonates w/ respiratory distress requiring mechanical ventilation and a UAC (N=39) | 1. PO (NPO + hypocaloric feedings of half strength premature formula 15-20 ml/kg/d starting at 48 hrs, intermittent gavage) (n=19)  2. NPO (the first 9 d of life) (n=20) | 1. Time to full enteric feedings  2. Days of PN and , feeding intolerance  3. Time to regain BW  4. ET days  5. UAC days  6. NEC | 1. Full enteric feedings at a mean of 16.1 d earlier in PO group  2. Days on PN, days of feeding intolerance requiring NPO (min 12h/d), time to regain BW, and NEC did not differ significantly between groups | Moderate |
| Terrin et al. Acta Paediatr. 2009;98:31-35. | Retrospective cohort | VLBW neonates w/ feed intolerance in the NICU (N=102) | 1. TPN (n=51)  2. PN plus minimal enteral feeds (10mL/kg/day) for 24 hr (n=51) | 1. Time to full enteric feedings  2. Late onset culture proven sepsis  3. Time to regain BW  4. LOS  5. NEC | 1. Infants receiving minimal enteral feeds achieved full enteral nutrition sooner than TPN only group (8 vs. 11 d) and had a lower incidence of sepsis episodes (15.7% vs. 33.3%)  2. No difference in incidence of NEC | Low |
| Prasad et al. J Neonatal Surg. 2018;7:21. | Prospective cohort | Neonates undergoing abdominal surgery (N=260) | 1. EEN (n=79)  2. NPO (n=181) | 1. LOS  2. SSI  3. Stress markers  4. Neo-PIRO  5. Intra-abdominal pressure grade  6. Tolerance of feeds  7. Time to first stool | 1. Lower SSI in EEN group (24% vs. 43%)  2. Shorter LOS in EEN group  3. Lower CRP levels and Neo-PIRO scores in EEN group  4. No significant differences in intraabdominal pressure or anastomotic leaks | Low |
| Jiang et al. Asia Pac J Clin Nutr. 2016;25:46-52. | Retrospective cohort | Neonates w/ gastric perforations undergoing surgical treatment (N=46) | 1. EEN (jejunal feeding tube w/ continuous EN infusions starting 48 h postop; n=24)  2. Control group (TPN; n=22) | 1. Time to first stool and first oral feed  2. LOS  3. Nutrition indices  4. Complications | 1. Time to first stool and LOS was significantly shorter in EEN group  2. Incidence of cholestasis and abdominal distension was significantly lower in EEN group  3. No difference in time to first oral feed or nutrition indices | Low |
| Morgan et al. Cochrane Database Syst Rev. 2015;15: CD001241. | Systematic Review  (9 studies) | Very preterm or VLBW infants | Delayed vs. earlier introduction of enteral feeds | 1. NEC  2. Time to full enteral feeds  3. Mortality | 1. Delayed initiation of feeds did not reduce the risk of developing NEC  2. Delayed initiation of feeds led to a longer time before full enteral feeds are established  3. No significant difference in all-cause mortality | High |
| Suri et al. Nutrition. 2002;18:380-382. | Prospective cohort | Newborns between the ages of 1 and 23 d (N=17) | Enteral feeding was started by postop day 2 in 14 cases. Period of feeding varied between 3 and 20 d, w/ a mean of 10.4 d in the surviving babies. | 1. Diagnosis, weight at presentation, age at presentation,  2. Onset of enteral feeding  3. Complications  4. Mortality | 1. No significant differences in weight loss or gain during the period of hospitalization (p=0.3)  2. 13/14 babies weighing >1.5 kg were discharged; the other baby died from neonatal septicemia.  3. Excellent results can be obtained w/ aggressive enteral nutrition support in newborns undergoing upper intestinal surgery | Low |
| Yamasaki et al. J Anesthes. 2011;25: 321-329. | Retrospective cohort | Neonates who underwent corrective surgery for congenital heart disease on CPB (N=51) | Fast track group: those operated on following introduction of a fast-track protocol  Control group: prior to the introduction of fast-track protocol | 1. Pain management  2. Time to extubation  3. Days of postop weight recovery  4. LOS  5. Mortality | 1. Fast track group has lower doses of intra-op fentanyl (p<0.001).  2. More pts in the fast track group were extubated within 24 h (p<0.001)  3. Fast track group had lower median # of days to postop weight recovery (p=0.003), and days in ICU (p=0.01)  4. Mortality in fast track was 0% vs. 17% in control (p=0.21). | Very low |
| Sharp et al. J Paediatr Child Health. 2000; 36:472-476. | Retrospective cohort | Population‐based study of gastroschisis in children born in 1986–96 in Western Australia (N=70) | N/A | 1. Duration of TPN  2. LOS | 1. Earlier commencement of enteral feeds was associated w/ shorter duration of TPN, w/ each day delay increasing TPN duration by 1.06 d (p=0.0001)  2. Earlier commencement of enteral feeds was associated w/ shorter LOS, w/ each day delay increasing LOS by 1.05 d (p=0.0001) | Very low |

**Breast Milk as First Nutrition**

**Use breastmilk as the first choice for nutrition.**

**Quality of Evidence Range:** Very low to high

| **Study** | **Type of Study** | **Population** | **Intervention** | **Outcome** | **Key Findings** | **Quality of Evidence** |
| --- | --- | --- | --- | --- | --- | --- |
| Lucas & Cole. Lancet. 1990;336:1519-1523. | Multi-center RCT  (5 sites) | Neonates within 48 hrs of birth w/ BW <1850g (N=926) | Two Studies:  Sole Diet Study  1. Donor breast milk  2. Preterm formula  Supplement Study  1. Maternal breast milk + 0-100% donor breast milk  2. Preterm formula | 1. NEC | 1. Exclusively formula-fed babies confirmed NEC was 6-10 times more common than those fed breast milk alone and 3 times more common in those fed formula plus breast milk  2. Type of human milk given did not affect the incidence of NEC  3. Delay in initiating feeds associated w/ a reduction in NEC for formula-fed infants but not for breast milk infants | High |
| Lucas et al. Lancet. 1980;1:1267-1269. | Prospective cohort | 6 day old healthy term infants (N=77) | 1. Breast-fed (n=43)  2. Bottle-fed cows’-milk formula (n=34) | 1. Hormone levels before feeds or 55, 90, 150 minutes after beginning of feed | Significant differences in alimentary hormone responses to feeding method:  1. Insulin (higher in formula-fed)  2. Motilin (formula-fed: higher basal level and significant drop at 150 mins)  3. EG (higher in formula-fed at 150 mins)  4. NT (higher basal level and more sustained response in formula-fed)  5. PP (higher in bottle-fed at 150 mins) | Very Low |
| Kleessen et al. Acta Paediatr. 1995;84:1347-1356. | Clinical trial Partial randomization (for which cow milk formula) | Healthy full-term infants (N=39) | 1. Breast Milk (n=20)  2. Cow Milk Formulas (n=19)  -MF1 (100% bovine casein; n=10)  -MF2 (whey/casein 60:40; n=9) | 1. Bacterial counts and pH of stool  2. Bacterial flora distribution | 1. Formulas were able to stimulate the growth of intestinal bifidobacteria, but were unable to suppress the growth of potential pathogenic organisms to the same extent as human milk | Very Low |
| Quigley et al. Cochrane Database Sys Rev. 2018;6: CD002971. | Systematic review  (11 studies) | Preterm or LBW infants (N~1800) | 1. Formula  2. Donor breast milk | 1. Growth and development  2. NEC | 1. Formula feeding associated w/ a significantly higher rate of weight gain, increase in length, and increase in head circumference  2. No significant long-term differences on growth or neurodevelopment  3. Formula fed infants: higher incidence of NEC (RR=1.87) | Moderate |
| Lambert et al. J Perinatol. 2007;27;437-443. | Retrospective cohort | Term or near-term neonates (N=430) | N/A | 1. NEC | 1. None of the neonates that were entirely breast-fed developed NEC  2. 53% of those that developed NEC had been exclusively formula fed, whereas only 13% of those that did not develop NEC were fed exclusively w/ formula (p=0.000)  3. Feeding by gavage was not more common among those that developed NEC | Low |

**Monitoring of Urinary Sodium**

**Monitor urinary sodium in all neonates with a stoma. Target urinary sodium should be greater than 30 mmol/L and exceed the level of urinary potassium**.

**Quality of Evidence Range:** Low

| **Study** | **Type of Study** | **Population** | **Intervention** | **Outcome** | **Key Findings** | **Quality of Evidence** |
| --- | --- | --- | --- | --- | --- | --- |
| Bower et al. J Pediatr Surg. 1988;23: 567-572. | Retrospective cohort | Neonates undergoing ileostomy construction for NEC or MI (N=11) | N/A | 1. Weight gain  2. Sodium intake  3. Caloric intake  4. Ileostomy output | 1. All 11 had metabolic abnormalities induced by ileostomy fluid loss  2. 7 out of 11 infants required supplementation w/ sodium  3. 6 infants initially lost or failed to gain weight despite adequate caloric intake; however, after sodium supplementation, all 6 gained weight and improved their metabolic acidosis | Low |
| Butterworth et al. J Pediatr Surg. 2014;49: 736-740. | Retrospective cohort | Infants ≤  12 months undergoing intestinal surgery w/ urine sodium measurement (N=39) | N/A | 1. Urine sodium  2. Serum sodium  3. Sodium intake  4. Ostomy output  5. Urine output  6. Weight | 1. 92% had sodium deficiency, 64% had severe deficiency  2. Infants receiving >50% of calories enterally:  - Urine sodium positively associated w/ weight gain (p=0.002)  - Those w/ urine sodium >30mmol/L has significantly higher weight gain than those w/ <30mmol/L | Low |
| Mansour et al. Pediatr Surg Int. 2014;30: 1279-1284. | Retrospective cohort | Infants <1yr that had an ileostomy, colostomy, or cystostomy (N=40) | N/A | 1. z-score  2. Urinary and serum sodium and potassium  3. Complications | 1. 75% had recorded urinary sodium of <10mM at some point  2. Urinary sodium positively associated w/ change in weight z-score (p<0.0003) (lower sodium correlated w/ larger growth deficit)  3. >10mM urinary sodium correlated w/ significant improvement in growth following surgery whereas <10mM at some time had significant growth failure (higher growth in >30mM than 10-30mM)  4. Ileostomy pts were the only ones to become severely malnourished postoperatively | Low |
| Schwarz et al. J Pediatr. 1983;102: 509-513. | Prospective cohort study | Neonates that underwent ileostomy in the first wk of life (N=7) | Sodium intake of 5-6 mEq/ kg/d 3 d before ileostomy closure | 1. Weight  2. Urine electrolytes  3. Fecal electrolytes | 1. For infants consuming 5 mEq Na/kg/d, GI Na losses were about ½ daily intake  2. Recommend that infants w/ ileostomy be provided w/ minimum of 6-10 mEq of Na/kg/d | Low |

**Mucous Fistula Refeeding**

**Use mucous fistula refeeding in neonates with enterostomy to improve growth.**

**Quality of Evidence Range:** Very low to moderate

| **Study** | **Type of Study** | **Population** | **Intervention** | **Outcome** | **Key Findings** | **Quality**  **of**  **Evidence** |
| --- | --- | --- | --- | --- | --- | --- |
| Lau et al. J Pediatr Surg. 2016;51:1914-1916. | Case control | Neonates w/ NEC. Mean GA 30.7 wks. (N=92) | Mucous fistula refeeding  (n=77) vs. no refeeding (n=15) | 1. Bowel end size discrepancy  2. Postop anastomotic leakage  3. PN-related cholestasis  4. Days on PN | 1. 25 vs. 53% (p=0.034)  2. 3 vs. 20% (p=0.029)  3. 42 vs. 73% (p=0.045)  4. 47 vs. 135 d (p=0.002) | Low |
| Wong et al. J Pediatr Gastro-enterol Nutr.  2004;39:43-45. | Case series | Neonates w/ short bowel syndrome. Median GA 31 wks. (N=12) | Mucous fistula refeeding | 1. Feasibility  2. Complications  3. Weight gain | 1. Successful in all pts  2. No complications  3. 18.9 g/day w/ refeeding vs. 10.5 g/day w/o refeeding | Very Low |
| Koike et al. J Pediatr Surg. 2016;51:390-394. | Case control | Neonates w/ enterostomy. Mean GA in intervention group 34.4 wks. (N=27) | Mucous fistula refeeding (n=13) vs. w/o refeeding (n=14) | 1. Rate of weight gain | 1. Significant difference in rate of weight gain (hazard ratio 18.1, 95% CI 0.61-1.81, p<0.0001) | Very Low |
| Al-Harbi et al. J Pediatr Surg. 1999;34:1100-1103. | Case series | Neonates w/ enterostomy. Mean GA 26 wks. (N=6) | Mucous fistula refeeding | 1. Weight gain w/ and w/o refeeding | 1. Improved average weight gain | Very Low |
| Gause et al. J Pediatr Surg. 2016;51:1759-1765. | Case control | Neonates w/ enterostomy. Median GA 34.6 wks vs. 30.9 (N=24) | With refeeding (n=13) vs. w/o refeeding (n=11) | 1. Days to ¨goal¨ enteral feeds  2. Time to weaning off PN  3. Time to ¨goal¨ enteral feeds after anastomosis | 1. 28 vs. 43 d (p=0.03)  2. 25 vs. 41 d (p=0.04)  3. 7.5 vs. 20 d (p<0.001) | Very Low |
| Haddock et al. J Pediatr Surg.  2015;50:779-782. | Case series | Neonates w/ enterostomy. Mean GA 35 wks. (N=23). | Mucous fistula refeeding | 1. Complications | 1. (n=4); three perforations, one bleeding, one death related to MFR. | Very Low |
| Richardson et al. J Pediatr Gastro-enterol Nutr. 2006;43:267-270. | Systematic review (5 studies) | Neonates. Mean GA 33 wks. (N=30) | Mucous fistula refeeding | 1. Weight gain  2. Complications | 1. Improved weight gain;  -2.36 – 21.06 g/day  2. No complications | Moderate |
