# Supplementary material for: Consensus Guidelines for Perioperative Care in Neonatal Intestinal Surgery: Enhanced Recovery After Surgery (ERAS®) Society Recommendations
Source: World J Surg. 2020 May 8;44(8):2482–92. doi: 10.1007/s00268-020-05530-1 (PMC7326795; doi:10.1007/s00268-020-05530-1)
Supplement: Supplementary file 5 — Complete guideline document with full reference list (DOCX 184 kb) [file 268_2020_5530_MOESM5_ESM.docx]

**Consensus guidelines for perioperative care in neonatal intestinal surgery:**

**Enhanced Recovery After Surgery (ERAS®) Society Recommendations**

Mary E. Brindle MD MPH^1^ and Caraline McDiarmid BSc^1,2^ (co-first authors), Kristin Short BA^1^, Kathleen Miller MSc^1^, Ali MacRobie^1^, Jennifer YK Lam MD^1^, Megan Brockel MD^3^, Mehul V. Raval MD MS^4^, Alexandra Howlett MD^5^, Kyong-Soon Lee MD MSc^6^, Martin Offringa MD PhD^6^, Kenneth Wong MD PhD^7^, David de Beer MD^8^, Tomas Wester MD PhD^9^, Erik D. Skarsgard MD MSc^10^, Paul W. Wales MD MSc ^11^, Annie Fecteau MD MHSc MSc^11^, Beth Haliburton RD^11^, Susan M. Goobie MD^12^, Gregg Nelson MD PhD^13^

^1^Department of Surgery, Alberta Children’s Hospital, Cumming School of Medicine, University of Calgary, 28 Oki Drive, Calgary, AB, T3B 6A8, Canada

^2^Department of Medicine, McMaster University, 1280 Main Street West, Hamilton, ON, L8S 4L8, Canada

^3^Department of Anesthesiology, Children's Hospital Colorado, 13123 E 16^th^ Avenue, Aurora, CO, 80045, United States of America;

^4^Division of Pediatric Surgery, Department of Surgery, Northwestern University Feinberg School of Medicine and Ann & Robert H. Lurie Children’s Hospital of Chicago, 225 E Chicago Avenue, Chicago, IL, 60611, USA

^5^Section of Neonatology, Department of Pediatrics, Alberta Children’s Hospital, Cumming School of Medicine, University of Calgary, 28 Oki Drive, Calgary, AB, T3B 6A8, Canada

^6^Division of Neonatology, Hospital for Sick Children, University of Toronto, 555 University Avenue, Toronto, ON, M5G 1X8, Canada

^7^ Department of Surgery, Li Ka Shing Faculty of Medicine, University of Hong Kong, 21 Sassoon Road, William M. W. Mong Block, Hong Kong, China

^8^Department of Pediatric Anesthesia, Great Ormond Street Hospital, Great Ormond Street, London, WC1N 3JH, United Kingdom

^9^Department of Pediatric Surgery, Karolinska University Hospital, Eugeniavagen 23, 171 76 Solna, Stockholm, Sweden

^10^Department of Pediatric Surgery, British Columbia Children’s Hospital, 4480 Oak Street, Vancouver, BC, V6H 3N1, Canada

^11^Division of General and Thoracic Surgery, Hospital for Sick Children, University of Toronto, 555 University Avenue, Toronto, ON, M5G 1X8, Canada

^12^ Department of Anesthesiology , Critical Care and Pain Medicine, Boston Children’s Hospital, 300 Longwood Avenue, Harvard University, Boston, MA, 02115, USA

^13^Department of Oncology, Cumming School of Medicine, University of Calgary, 1403 29 Street NW, Calgary, AB, T2N 2T9, Canada

Short Title: ERAS® Guidelines for Neonatal Intestinal Surgery

Keywords: ERAS, Neonatal, Intestinal

Grant Support: The Brian and Brenda MacNeill Chair in Pediatric Surgery, Alberta Children’s Hospital Foundation

Conflicts of Interest: Author Gregg Nelson (author 20) has a nonfinancial relationship with the ERAS® Society as their secretary.

Word Count: 5191

Mary E Brindle MD MPH

Department of Surgery and Community Health Sciences

Cumming School of Medicine, University of Calgary

28 Oki Drive

Calgary, Alberta, T3B 6A8

Canada

Phone: (403) 955-2848

Fax: (403) 955-7634

MaryEBrindle@gmail.com

Abstract

Background: Enhanced Recovery After Surgery (ERAS®) guidelines integrate evidence-based practices into multimodal care pathways that have improved outcomes in multiple adult surgical specialties. There are few pediatric ERAS® guidelines, none of which have focused on neonates. We have used a rigorous approach to create an ERAS® guideline designed to enhance quality of care and reduce perioperative adverse events in neonatal intestinal resection surgery.

Methods: A guideline generation group, comprised of researchers, methodologists, and clinical stakeholders, defined the scope, population, and guideline topics using the Delphi method. A series of focused systematic reviews was supplemented by targeted searching and expert identification. Evidence-supported recommendations and evidence tables were created by expert subgroups and rated based on necessity for inclusion. Revised recommendations were further reviewed, and inclusion/exclusion was determined through consensus. Final recommendations were assessed for evidence quality and recommendation strength according to the GRADE approach. Parental input was attained throughout the process.

Results: 14 topics, and 18 subtopics, were identified and reviewed by members of the guideline generation group. Systematic literature searches identified 2909 titles and abstracts. Final recommendations were iteratively developed and ranged from communication strategies, to parental roles, feeding regimens and antibiotic use. Topics with poor-quality and conflicting evidence were eliminated (e.g., skin preparation). Several recommendations were combined (e.g., pain management strategies). The quality of supporting evidence was variable. 17 final recommendations are included in the proposed guideline.

Conclusion: Using GRADE methodology, we have developed a comprehensive, evidence-based ERAS® guideline for neonates undergoing intestinal resection surgery. This first neonatal ERAS® guideline has the potential to bridge the gap between evidence and current neonatal surgical practice. This guideline, and its creation process, provides a foundation for future ERAS® guideline development, and can ultimately lead to improved perioperative care across a variety of pediatric surgical specialties.

Introduction

Enhanced Recovery After Surgery (ERAS®) guidelines are multidisciplinary, holistic tools designed to improve patient outcomes by encouraging the delivery of standardized, evidence-based, collaborative care throughout the surgical journey [1-9]. ERAS® guidelines follow a specific structure and are created through a rigorous process of development, based on systematic reviews of evidence and grading of evidence and recommendations [10]. They differ from many other surgical care pathways and guidelines in that they are multidisciplinary and multimodal and cover the entire surgical journey through synergistic recommendations aimed at reducing surgical stress and improving patient experience and outcomes [10].

ERAS® guidelines were first established in adult colorectal surgery and have subsequently been adapted for multiple other surgical subspecialties [11]. The implementation of these guidelines has led to improvements in outcomes; including reductions in complication rates, length of stay (LOS), healthcare costs, as well as improved patient, family, and staff satisfaction [6-8, 12-14]. Despite these successes, ERAS® has made few inroads into pediatric surgery, and none into neonatal surgery. To date, the majority of studies examining ERAS® guidelines for children have focused on a few recommendations with a primary focus on decreasing hospital LOS [4]. Implementation of these “fast track” protocols have, nonetheless, been associated with reductions in surgical infections, readmission rates, LOS, reoperation, and resource utilization [4, 15-18]. A more extensive ERAS® approach has been explored within a number of recent pediatric studies adopting adult ERAS® guidelines and showing considerable opportunity for benefit [19-24].

Neonates could greatly benefit from the principles of ERAS®. Neonatal surgical patients experience considerable variability in their perioperative care and suffer high rates of preventable adverse events. For example, surgical site infections (SSIs) occur in up to 13.5% of thoracic and abdominal neonatal surgeries; more than twice the rate reported in adults [25]. SSIs in these infants are associated with poor growth, longer hospital stays, increased rate of reoperation, and increased mortality [25-28].

The vastly different needs and challenges of neonatal surgical patients warrants the development of tailored care pathways. Neonatal ERAS guidelines must consider unique neonatal physiology such as nutritional requirements for growth and healing, exquisite sensitivity to fluid balance, temperature instability, and a markedly different immune response to surgical stress [28-31]. The social needs of neonatal patients are also unique, with an important role played by the neonatology team and parents [32-33].

Our International team has collaborated to develop the first ERAS® guideline for perioperative care in neonatal surgery. We followed a rigorous, evidence-based, and consensus-driven process to create a standardized and scalable guideline for neonatal intestinal resection surgery. In addition to establishing a comprehensive evidence base of published research, we have integrated parental and clinical stakeholder involvement throughout the process to develop a user-sensitive implementation strategy that will optimize impact and acceptance. This study is the first pediatric ERAS® guideline to be endorsed by the ERAS® Society.

Methods

The process that has been used to create the neonatal ERAS® guideline uses elements of knowledge synthesis, consensus generation and rigorous rating of evidence and recommendations. The details of our approach have been published but are summarized below [34].

Establishment of a multidisciplinary team

A guideline development committee (GDC) consisting of 11 members, and a group of subject matter experts (an additional 5 members) was assembled to create this guideline. The GDC members included pediatric surgeons, pediatric anesthesiologists, and neonatologists. Subject matter experts added additional expertise within each of the guideline topics. Parent representatives were consulted at multiple points during guideline development including topic and subtopic creation, during the full-day workshop and prior to final refinement of the tool for implementation.

Scope determination

From June to July 2017, the GDC convened through several teleconferences to discuss the scope of the guideline. A modified Delphi method was used to reach consensus regarding the target population, conditions for inclusion, and the general ERAS® topics. The target population was determined to be term neonates (infants born at or after 37 weeks) without major comorbidities undergoing intestinal surgery within the first 4 weeks of life. Conditions for inclusion were intestinal resections, including stomas. Complex surgical conditions were excluded including necrotizing enterocolitis, abdominal wall defects and massive bowel resections leading to short gut syndrome. A total of 14 topics were identified for areas of recommendation development (Online Resource 1) [34].

Literature search

Each guideline committee member was assigned 1-2 topics to review based on experience and preference. For each topic, a systematic search strategy was developed in conjunction with a research librarian (Online Resource 2). The searches were performed in December 2017 utilizing the databases MEDLINE and CINAHL. Guideline committee members supplemented these searches with further targeted literature searches, citation searching, review of personal archives, and discussion with content experts [34].

Study selection and data synthesis

Abstracts were screened using Rayyan QCRI, a web-based systematic review application. Meta-analyses, systematic reviews, randomized controlled studies, non-randomized controlled studies, observational cohorts and case series were all considered. Case studies and expert opinion were not included. Potentially relevant articles meeting eligibility criteria were reviewed in full-text. Several of the 14 ERAS® topics had subtopics developed through deductive and inductive means. One or more recommendations were drafted for each ERAS® topic [34]. In addition, a summary of the supporting evidence was tabulated, with the Grading of Recommendations, Assessment, Development and Evaluation (GRADE) [35] approach being used to assess the quality of evidence supporting each recommendation. The GRADE approach uses a 4-tiered system ranging from ‘very low’ to ‘high’ quality based on risk of bias, imprecision, inconsistency, indirectness, and publication bias [35]. This rigorous approach allows appropriate high quality evidence from a different patient population to be used to develop recommendations; but also requires that the quality be appropriately rated down based on its indirectness.

Quality assessment and grading

The RAND/UCLA Appropriateness Method, a two-round modified Delphi, was used to review and select the recommendations [36]. In the first round of review, the drafted recommendations and supporting evidence tables were circulated to guideline committee members. Using an online survey, the guideline committee members were asked to rate the clarity and necessity for inclusion and provide feedback. The second round of review consisted of a full-day workshop in Toronto, Ontario, Canada in July 2018, where the recommendations were discussed and further revised by the committee. Recommendations were again voted on for inclusion in the guideline. Items with a consensus for inclusion were assessed for quality of the aggregate data and the strength of the recommendation according to the GRADE approach (see Tables 1a and 1b) [35]. The strength of the recommendation was determined through consensus of the guideline committee using the GRADE approach and based on consideration of the quality of evidence, published standards and national guidelines as well as potential desirable and undesirable consequences of the recommendation [35]. Recommendations were rated as ‘strong’ or ‘weak’. All recommendations were further reviewed by small teams of experts (i.e. transfusion medicine specialists, nurse educators) and potential guideline users (bedside nurses, clinician groups) to ensure feasibility and acceptability.

Results

A total of 3514 total texts were reviewed, with 2909 texts identified via initial systematic searches and 605 identified through additional targeted searches, citation searches, and expert identification. The details of the search strategies can be found in Online Resource 2 and the PRISMA diagrams for these searches can be found in Online Resource 3. Guideline committee members reviewed the articles and submitted related recommendations for the ERAS® guideline. This resulted in a total of 36 preliminary recommendations.

Based on the results of the consensus meetings, recommendations were eliminated (e.g. laparosopic approach, skin preparation) due to poor-quality or conflicting evidence. Several other recommendations were re-worded or combined.

The final ERAS® guideline has 17 recommendations (Table 2) (Figure 1). Overall, 116 academic articles were used to support the recommendations (Online Resource 4). In general, the quality of evidence of these papers was relatively low. 68.1% (n=81) of the papers had a rating of Very Low (23%; n=27) or Low (45%; n=54), 22% (n=26) had a rating of Moderate, and 10% (n=12) had a rating of High.

Evidence Base And Recommendations

Surgical Practices

In the setting of intestinal atresia, pediatric surgeons must frequently decide between creation of a stoma or performing primary anastomosis. Very low quality evidence comparing primary and secondary anastomosis in neonates with intestinal atresia, demonstrates that primary anastomosis is associated with a reduction in LOS, time of PN, readmissions, and the need for reoperations [37-41]. However, the likelihood for primary anastomosis is higher in neonates with uncomplicated atresia, likely introducing selection bias. As a result, the recommendation has been limited to neonates with uncomplicated atresia.

| *Recommendation:* | **Perform primary anastomosis as the first choice in patients with uncomplicated intestinal atresia.** |
| --- | --- |
| *Evidence Quality:* | Very low |
| *Recommendation Strength:* | Weak |

Antimicrobial Prophylaxis

Antibiotics within 60 minutes

The quality of evidence regarding antibiotic prophylaxis for neonatal surgery is low. Given the understanding of neonatal pharmacodynamics, the comparatively high SSI rate in neonatal intestinal surgery, and the relatively immunocompromised state of neonates, recommendations were deemed important and reasonable to extrapolate from the adult literature [42]. High quality evidence demonstrates decreased SSI rates in adult intestinal surgery patients provided with appropriate, well-timed preoperative antibiotics [42]. Studies of neonatal pharmacokinetics suggest that the less than 60 minute time frame for prophylactic dosing would also be effective for neonates [43]. Re-dosing schedules differ based on the drug’s half-life and pharmacokinetic properties. These recommendations are additionally supported by international societies and practice guidelines [44-45].

| *Summary and Recommendation:* | **Administer appropriate preoperative antibiotic prophylaxis within 60 minutes prior to skin incision.** |
| --- | --- |
| *Quality of Evidence:* | Low |
| *Strength of Recommendation:* | Weak |

Duration of postoperative antibiotics

There are few studies investigating the optimal duration of perioperative antibiotic prophylaxis after neonatal intestinal surgery. Institutional practice guidelines often suggest that the duration of prophylactic antibiotics should not exceed 24 hours [46]. Low quality studies show no difference in SSI rates when prophylactic antibiotics were given for less than 24 hours as compared to greater than 24 hours [47-48]. High quality adult literature suggests that a single preoperative dose of antibiotics is sufficient for prophylaxis in most patients, however, similar evidence to support the use of a single preoperative dose of prophylactic antibiotics in the neonatal population is lacking [45-47]. Given the significantly higher rate of SSIs in neonates, general practice has been to provide longer periods of prophylactic antibiotics as demonstrated in a survey study where patients received inconsistent and prolonged prophylactic antibiotic courses with some extending beyond 1 week [49]. Antibiotic administration, however, is not without harm. The impact of antibiotic exposure on the neonatal microbiome, the increasing risk of invasive candida infections, and the emergence of resistant organisms within NICUs underlie the value of antibiotic stewardship [50]. The recommendation for limited duration (< 24 hours) of postoperative antibiotics is made in the context of prophylactic antibiotics. In the setting of a documented infection or wound contamination (higher wound class), an appropriate therapeutic antibiotic regimen should be pursued.

| *Summary and Recommendation:* | **Discontinue postoperative antibiotics within 24 hours of surgery, unless ongoing treatment is required.** |
| --- | --- |
| *Quality of Evidence:* | Low |
| *Strength of Recommendation:* | Weak |

Preventing Intraoperative Hypothermia

Due to large surface area to volume ratio and poor thermoregulation, neonates are at high risk of surgical hypothermia (<36.5°C) [51-52]. Routine use of temperature monitors, however, is frequently overlooked and neonates are at risk of hypothermia during surgery as well as during transportation. In adult perioperative patients, hypothermia has been associated with an increase in the incidence of morbid cardiac outcomes, surgical blood loss, and SSIs [53]. Hypothermic infants have been shown to suffer more respiratory adverse events and require more support interventions than their non-hypothermic counterparts [54]. Implementation of hypothermia bundle has resulted in a significant reduction (53-75%) of perioperative hypothermia [55-56]. Neonates appear to be most at risk of hypothermia in the OR.^49^ Despite a relatively low quality of evidence, the potential risks of hypothermia in neonates warrants a strong recommendation.

| *Summary and Recommendation:* | **Continuously monitor intraoperative core temperature and take pre-emptive measures to prevent hypothermia (<36.5°C) and maintain normothermia.** |
| --- | --- |
| *Quality of Evidence:* | Low |
| *Strength of Recommendation:* | Strong |

Perioperative Fluid Management

Perioperative fluid management in neonates aims to maintain normal physiological state, tissue perfusion, metabolic function, and acid-base-electrolyte status. Perioperative fasting times should be as short as possible to prevent patient discomfort, dehydration, and ketoacidosis [57]. Isotonic solutions with glucose are recommended for intraoperative fluid administration. Monitoring clinical status, response to fluids, blood glucose, blood gases and electrolytes is a key part of intraoperative care [58]. Both hyperglycemia and hypoglycemia have been documented in neonates with different fluid regimens in the OR [59]. Glucose containing fluids may decrease the incidence of intra-operative hypoglycemia but higher concentrations may contribute to hyperglycemia [59]. A target glucose range of 3.3 to 7 mmol/L is suggested based on definitions of hypo- and hyper-glycemia in the neonatal population beyond three days of life [60-61] . However, similar definitive target glucose values for an anesthetized infant for whom one cannot effectively monitor for signs and symptoms of hypoglycemia do not exist in the literature. As such, clinicians may choose to target a slightly higher range for infants under anesthesia, while also recognizing that levels above 8 mmol/L may contribute to future detrimental effects on neurodevelopment. Hypotonic IV fluids should not be used as they heighten the risk for hyponatremia in at risk children [62-63]. Colloids are only recommended to recover normovolemia and to avoid fluid overload when crystalloids alone are not sufficient and blood products are not indicated. Despite the moderate quality of evidence supporting this recommendation, there is a paucity of studies that clearly demonstrate the downstream effects and therefore, specific regimens cannot be suggested and the strength of the overall recommendation is weak.

| *Summary and Recommendation:* | **Use perioperative fluid management to maintain tissue perfusion and prevent hypovolemia, fluid overload, hyponatremia, and hyperglycemia.** |
| --- | --- |
| *Quality of Evidence:* | Moderate |
| *Strength of Recommendation:* | Weak |

Perioperative Analgesia

Pain management with acetaminophen

Acetaminophen, administered via the enteral or intravenous (IV) route, is widely used to treat pain in neonates and infants, and is part of a multimodal analgesia regime aimed at reducing opioid exposure after major surgery. Multiple studies, including an RCT of neonates and infants following major non-cardiac surgery, indicate that IV acetaminophen reduces postoperative morphine consumption in neonates when compared with other regimens [64]. These findings have not been replicated with rectal acetaminophen. Safe dosing in neonates is dependent on a thorough understanding of patient pharmacokinetics [65]. When IV acetaminophen is not available, rectal acetaminophen should be given. Despite concerns about the hepatic effects of IV acetaminophen, low doses are well tolerated in term neonates and have a good safety profile when used for a limited period (48-72h) [66]. Although current evidence suggests that the use of acetaminophen in therapeutic doses is safe with regard to short-term outcomes, longer-term safety aspects need more clarification [65-67]. Acetaminophen should be given regularly (not prn) during minor painful procedures in neonates with strict adherence to the recommended dose, dosing interval, and maximum allowable daily dose.

| *Summary and Recommendation:* | **Unless contraindicated, administer acetaminophen regularly during the early postoperative period (not on an “as needed” basis) to minimize opioid use.** |
| --- | --- |
| *Quality of Evidence:* | High |
| *Strength of Recommendation:* | Strong |

Opioid use

Morphine, the most commonly used opioid in neonates, is effective in treating postoperative pain following major surgery [68]. However, pharmacokinetic differences lead to less predictable clinical effects in neonates as compared to older children. These differences result in an increased variability in plasma concentrations of morphine and its metabolites in neonates [69]. As a result, reduced doses and increased dosing intervals are necessary in order to avoid accumulation and the risk of sedation and respiratory depression [70].

Postoperatively, morphine can be administered by continuous infusion, intermittent bolus dosing, or via a nurse-controlled analgesia (NCA) regime. In two RCTs of infants following major surgery, the efficacy of continuous vs. bolus morphine was similar, although slightly higher pain scores were observed in infants receiving regular bolus morphine [68, 71].

The use of morphine is not without potential adverse effects, as the therapeutic window between analgesia and respiratory depression is narrow. Higher rates of opioid related respiratory depression are seen in neonates as compared to older children [70]. Other important adverse effects include hypotension and decreased gastrointestinal motility [72]. Although morphine is the gold standard opioid, fentanyl is increasingly being used within the intensive care setting. While further evidence regarding safety and efficacy is required, a large double-blind RCT indicated that fentanyl may be superior to morphine for short-term analgesia in neonates [72].

Postoperatively, an opioid sparing, multimodal analgesia strategy should be used. The lowest dose of opioid should be given for the shortest possible time. All neonates receiving opioids should be managed in an appropriate environment with continuous pulse oximetry and monitoring of other vital signs and regular assessment of pain scores.

| *Summary and Recommendation:* | **Use an opioid-limiting strategy is recommended in the postoperative period. Manage breakthrough pain with the lowest effective dose of opioid with continuous monitoring.** |
| --- | --- |
| *Quality of Evidence:* | Moderate |
| *Strength of Recommendation:* | Strong |

Standard analgesia protocol

The evolution of ultrasound-guided regional anesthetic techniques and the use of regular acetaminophen has allowed for reduced exposure of infants to high doses of opioids and other anesthetic agents and helped to reduce opioid and anesthetic complications [73]. Epidural analgesia, when combined with general anesthesia, decreases respiratory complications and shortens the time to full bowel function [74-75]. Regional blocks including transversus abdominus plane (TAP) blocks and caudal blocks avoid some of the risks of epidurals while achieving good pain control [76-77]. In appropriate neonates, the use regional anesthesia and regular (not prn) acetaminophen is recommended and may reduce the need for intraoperative and postoperative narcotics. In addition, regional and spinal blocks can reduce the exposure of neonates to volatile anesthetic agents, diminishing potential neurotoxic effects.

| *Summary and Recommendation:* | **Use regional anesthesia and acetaminophen perioperatively in combination with general anesthesia. Multi-modal strategies including regional techniques should be continued postoperatively.** |
| --- | --- |
| *Quality of Evidence:* | High |
| *Strength of Recommendation:* | Strong |

Lingual sucrose/dextrose

Neonates experience many painful procedures daily in the ICU. Numerous interventions to manage neonatal pain have been evaluated [78-81]. Many studies have demonstrated the feasibility and efficacy of lingual sucrose/dextrose as an analgesic in neonates. A 2016 systematic review of 74 RCTs demonstrated high quality evidence of the benefits of oral sucrose in preterm and term infants undergoing heel lance, venipuncture, and intramuscular injections [82]. Lower quality studies showed sucrose to be of some benefit for other interventions such as arterial puncture, insertion of naso/orogastric tubes, bladder catherization, etc.

Two randomized double-blind placebo-controlled clinical trials demonstrated significantly lower pain scores with sucrose during nasogastric tube placement compared with the placebo [83-84]. Infants randomized to sucrose treatment also demonstrated very little change in heart rate during NGT insertion compare to those randomized to receive placebo [84]. Due to the low morbidity and ease of incorporation into practice, we recommend lingual sucrose/dextrose to reduce pain during naso/orogastric tube placement and prior to other minor painful procedures.

| *Summary and Recommendation:* | **Provide lingual sucrose/dextrose to reduce pain during naso/orogastric tube placement and other minor painful procedures.** |
| --- | --- |
| *Quality of Evidence:* | High |
| *Strength of Recommendation:* | Strong |

Optimal Hemoglobin

Neonates are physiologically different from infants and young children and therefore require a distinct set of hemoglobin thresholds and transfusion guidelines. Neonates with anemia are at high risk of mortality, however due to the lack of a universally accepted definition of anemia in neonates and the wide reference range of acceptable hematocrit values for this age group, it is challenging to determine an optimal safe hemoglobin threshold [85]. This is confounded by the fact that neonates have varying hemoglobin levels and types and a limited ability to tolerate physiologic stress. Current evidence regarding optimal hemoglobin thresholds in neonates is controversial and evolving. Most evidence-based sources recommend a relatively restrictive hemoglobin threshold in term neonates given that no significant differences in short term outcomes have been found comparing restrictive and liberal strategies. These guidelines take into account age and respiratory status. Given that a neonates’ total blood volume can often be less than 100 mL, methods to decrease blood loss should be considered including measures to minimize both blood sample volume and testing frequency. Initial laboratory testing may be performed on a cord blood sample as one strategy to decrease phlebotomy blood losses. When indicated, red blood cell transfusions should be single donor, leukocyte depleted, irradiated, and fresh [86]. Recommendations for the optimal hemoglobin threshold for term neonates is based on expert consensus guidelines and a small number of indirect, moderate quality, randomized trials [85, 87-88].

| *Summary and Recommendation:* | **Restrict transfusions to maintaining HgB >/=90 (9 g/dL for a term neonate with no oxygen requirement. Term neonates within the first week of life, intubated or with an oxygen requirement should be transfused to maintain a HgB >/=110 (11 g/dL).** |
| --- | --- |
| *Quality of Evidence:* | Low |
| *Strength of Recommendation:* | Weak |

| *Summary and Recommendation:* | **Use written transfusion guidelines and take into account not only a target hemoglobin threshold, but also the clinical status of the neonate and local practices.** |
| --- | --- |
| *Quality of Evidence:* | Low |
| *Strength of Recommendation:* | Weak |

Perioperative Communication

Standardization of perioperative communication and care processes can result in a reduction in adverse patient outcomes [11]. Both pre- and post-operative communication with a structured process and protocol have been found to be helpful in safeguarding continuity of care and improving staff communication [89-93]. A systematic review of post-operative handovers found that successful elements in these processes include: a) use of checklists, b) completion of urgent tasks prior to handover, c) only patient-specific discussions allowed during verbal handover, d) presence of all relevant team members at handover, and e) provision of training in team skills and communication [94]. Staff engagement and consistent teamwork have been shown to be the most important factors for promoting a safe surgical environment [95]. Due to the potential adverse effects of miscommunication, interdisciplinary team participation by the entire pre-, intra-, and post-operative team in a structured perioperative communication process should be implemented [95].

| *Summary and Recommendation:* | **Implement perioperative multidisciplinary team communication with a structured process and protocol (“pre- and post-operative huddle”) utilizing established checklists.** |
| --- | --- |
| *Quality of Evidence:* | Moderate |
| *Strength of Recommendation:* | Strong |

Parental Involvement

Parental stress is common in the NICU. As educational interventions for parents have not been shown to significantly reduce stress [96-98], there has been a focus on improving parent-provider communication. Parent-provider partnerships in the delivery of care, including improved communication, may help to mitigate parental anxiety. Particular areas of care that require special attention to knowledge transmission from provider to parent include perioperative risks of surgery and anesthetics as well as potential long-term consequences. Family centred rounds and parental inclusion in ward rounds have been shown to improve patient outcomes and parental satisfaction [99-101]. Providing families with information regarding communication (e.g., contact cards) also resulted in increased family satisfaction [102]. Additionally, the use of technology, such as smartphone-texts and webcams, can enhance communication with parents [103-104]. Parental involvement should be individualized, and special consideration should be provided for patients speaking different languages, of different ethnicities, teens, and fathers; each of who have different support needs [105-107].

Parental involvement in the preterm infant’s discharge planning is helpful in ensuring a successful transition to home. While current literature includes primarily qualitative studies in non-surgical populations, the process issues have relevance for the neonatal surgical population. The discharge experience has been perceived by parents as feeling rushed and confusing with inconsistent communication [108-109]. To better prepare parents for discharge after surgery, teaching parents necessary skills should be initiated early and continued throughout their hospital stay [96-97, 110]. This iterative training process should continue up until the time of the infant’s discharge. Providing parents with educational opportunities throughout the hospital stay increases parental knowledge, confidence, and satisfaction, and has been associated with improved infant developmental outcomes, increased compliance with well-baby checks, and reduced emergency room visits [96, 111-112]. Written materials, audiovisual aids, and simulation have all been found to be helpful by parents [96-97, 112-113]. Parent-partnered decision-making and communication is key to achieving the best outcomes for families after discharge.

| *Summary and Recommendation:* | **Facilitate hands on care and purposeful practice by parents that is individualized to meet the unique needs of parents early during the admission. Sustain these to build the knowledge and skills of parents to take on a leading role as caregivers and facilitate their readiness for discharge.** |
| --- | --- |
| *Quality of Evidence:* | High |
| *Strength of Recommendation:* | Strong |

Postoperative Nutritional Care

Early feeding

There is considerable evidence to support early enteral feeding in post-surgical neonates. In prospective trials and cohort studies, neonates that were fed early achieved a significantly shorter time to first stool, a significantly shorter LOS, and decreased surgical site infections with no increase in anastomotic leaks [114-115]. Additionally, in very low birthweight infants, the early introduction of low volume hypocaloric feeds showed no increased incidence of necrotizing enterocolitis (NEC) and a decreased incidence of sepsis [116-118]. Although there is relatively high quality evidence supporting this recommendation, variable clinical situations may support a delay in feeding so the recommendation was determined to be weak.

| *Summary and Recommendation:* | **Start early enteral feeds within 24-48 hours after surgery when possible. Do not wait for formal return of bowel function.** |
| --- | --- |
| *Quality of Evidence:* | High |
| *Strength of Recommendation:* | Weak |

Breast milk as first nutrition

Breast milk is recognized as a resource friendly, optimal feeding choice for healthy term infants, and the benefits have been widely reported. In the post-surgical population, where feeding intolerances are common, breast milk is typically well tolerated and the presence of immunoglobulin, prebiotics, and growth factors are thought to improve intestinal maturation and adaptation [119-121]. The protective effect of breast milk on the development and recurrence of NEC in preterm and low birth weight infants is well known [122-123]. Breast milk consumption promotes the development of beneficial fecal flora and supresses the growth of potential pathogenic organisms in term infants [124].

| *Summary and Recommendation:* | **Use breastmilk as the first choice for nutrition.** |
| --- | --- |
| *Quality of Evidence:* | High |
| *Strength of Recommendation:* | Strong |

Monitoring of urinary sodium

Infants who undergo stoma creation commonly suffer from sodium depletion [125-126]. In these patients, lower urinary sodium is associated with larger growth deficits whereas no relationship is seen between growth and serum sodium levels [126]. Inadequate urine sodium concentration is associated with slower weight gain in infants undergoing intestinal surgery. Surgical neonates with a stoma demonstrating slow weight gain, despite the provision of presumed adequate calories, should have their urine sodium levels monitored.

Sodium supplementation to maintain urinary sodium >30 mmol/L improves overall growth in surgical neonates [125]. The value of using urine sodium to guide sodium replacement includes its responsiveness in relation to sodium supplementation and the ability to avoid excessive blood draws. The frequency of monitoring depends on whether active replacement is ongoing. Published institutional protocols can guide this process [125]. The relatively low quality of evidence supports a case-by-case approach when supplementing surgical neonates with sodium and points to the necessity of ongoing monitoring of urinary sodium levels.

| *Summary and Recommendation:* | **Monitor urinary sodium in all neonates with a stoma. Target urinary sodium should be greater than 30 mmol/L and exceed the level of urinary potassium.** |
| --- | --- |
| *Quality of Evidence:* | Low |
| *Strength of Recommendation:* | Weak |

Mucous Fistula Re-feeding

Enteral nutrition is required in all neonates for optimal healing and growth. In neonates with enterostomy, mucous fistula refeeding is used to accelerate this process. Studies evaluating mucous fistula refeeding in neonates tend to be indirect and of low to moderate quality [127-133]. Acknowledging these limitations, mucous fistula refeeding has been associated with improved weight gain, shorter time of parenteral nutrition (PN), decreased bowel end size discrepancy at time of anastomosis, decreased risk for postoperative anastomotic leakage, and less PN-related cholestasis in neonates with enterostomy [127-131, 133]. Complications are rare; however, the risk of bowel perforations and potential death lends caution [132]. The decision to proceed with mucous fistula feeding necessitates careful patient selection (term infants with a healthy and healed distal stoma with small bowel distal to the mucous fistula, while specific contraindications to mucous fistula refeeding include clinical instability, an unhealthy or atretic mucous fistula or bowel obstruction distal to the mucous fistula). Additionally, the safe practice of mucous fistula refeeding should include ongoing monitoring, thorough nursing education and, ideally should follow a standard institutional protocol created with multidisciplinary stakeholder input.

| *Summary and Recommendation:* | **Use mucous fistula refeeding in neonates with enterostomy to improve growth.** |
| --- | --- |
| *Quality of Evidence:* | Moderate |
| *Strength of Recommendation:* | Weak |

Excluded Items and Weak Evidence

This guideline outlines ERAS® recommendations for the perioperative management of neonatal patients undergoing intestinal resection surgery and is based on the best available evidence. In some instances, good quality data was not available (urinary catheter use) or data was conflicting (chlorhexidine vs. poviodine based skin preparation) thus preventing the development of recommendations.

Many recommendations within this guideline are supported by weak evidence. In some of these cases supporting evidence is indirectly inferred from high quality evidence in other populations (e.g. preoperative antibiotic recommendation), in other cases, recommendations were judged to be important as potential benefits were judged to considerably outweigh harms (e.g., prevention of hypothermia recommendation). Finally, in some cases, weak evidence was strongly bolstered by national guidelines and standards (e.g., transfusion recommendations [87-88]

Conclusion

Enhanced Recovery After Surgery (ERAS®) guidelines integrate evidence-based practices into multimodal care pathways in order to optimize patient recovery following surgery [11]. These patient-focused guidelines function to improve surgical outcomes and reduce undesirable care variations through a team-based approach [2, 11, 15].

A neonatal ERAS® guideline is necessarily different from existing adult guidelines due to neonatal physiological differences as well as the unique nature of the care teams involved in neonatal surgery. We adapted the GRADE process for evaluating evidence and determining the strength of recommendations to improve the care of neonates undergoing intestinal resection. This article presents the evidence base and recommendations for 17 items within the multimodal perioperative care pathway. These recommendations represent the best-available evidence in the literature evaluated and reviewed by expert teams. Although ERAS® is new to pediatric surgery, we anticipate that its role will grow in pediatric surgical quality improvement.

The development of this ERAS® care pathway has uncovered the need for higher quality data to guide the management of neonatal surgical patients. Although recommendations are supported by existing data, much of this data is of low to moderate quality. Each recommendation within this guideline has been reviewed by teams of users and experts within the field both to ensure that guidelines are feasible but also to ensure recommendations generated from low and moderate quality evidence maximize the opportunity for benefit and minimize the likelihood of harm. The guideline will undergo regular review and higher quality data will be used as it becomes available to further develop recommendations, improve clinician uptake and optimize patient outcomes [10]. In the meantime, standardizing practices will decrease variability in care and has a significant likelihood of improving patient outcomes. Successful adoption of ERAS® care pathways requires a strong implementation strategy that encourages broad collaboration and engagement. Effective implementation will require an integrated system of auditing, local leadership buy-in, consideration of local contexts, as well as a commitment to continuous quality improvement in order to develop a tailored approach. This iterative and longitudinal approach to enhanced recovery and patient safety measures will ultimately improve the perioperative management of neonatal patient undergoing intestinal surgery.

Acknowledgements: Ashley Jones, Rita Visconti and Francine Buchanan, parents of neonatal surgical patients, provided invaluable input and advice.


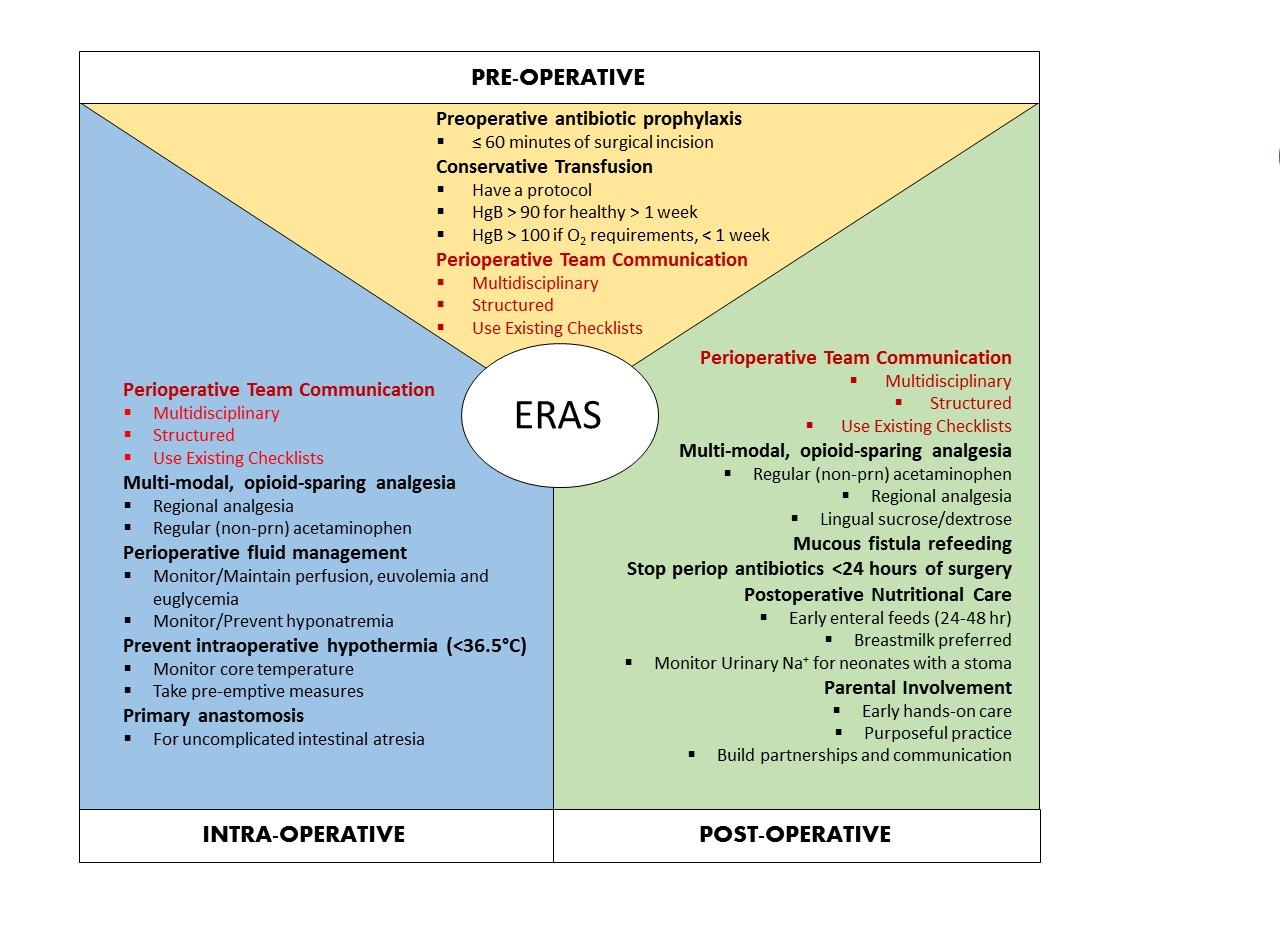


**Fig. 1** Elements of the ERAS® Approach for Neonatal Intestinal Resection Surgery. Refer to Table 2 for greater detail on each recommendation.

Supplementary Material Captions

**Online Resource 1.** Topics identified by guideline committee for ERAS® recommendation development

**Online Resource 2.** Systematic search strategies used for each topic

**Online Resource 3.** PRISMA diagrams outlining evidence screening process

**Online Resource 4.** Summary of evidence and quality of evidence used in support of each recommendation

References

1. Ljungqvist O (2014) ERAS—Enhanced Recovery After Surgery: Moving Evidence-Based Perioperative Care to Practice. J Parenter Enter Nutr 38(5):559-566

2. Gramlich LM, Sheppard CE, Wasylak T, et al (2017) Implementation of Enhanced Recovery After Surgery: a strategy to transform surgical care across a health system. Implement Sci 12(1):67

3. Nelson G, Kiyang LN, Crumley ET, et al (2016) Implementation of Enhanced Recovery After Surgery (ERAS) Across a Provincial Healthcare System: The ERAS Alberta Colorectal Surgery Experience. World J Surg 40(5):1092-1103

4. Shinnick JK, Short HL, Heiss KF, et al (2016) Enhancing recovery in pediatric surgery: a review of the literature. J Surg Res 202(1):165-176

5. Roulin D, Najjar P, Demartines N (2017) Enhanced Recovery After Surgery Implementation: From Planning to Success. J Laparoendosc Adv Surg Tech 27(9):876-879

6. Sibbern T, Bull Sellevold V, Steindal SA, et al (2017) Patients’ experiences of enhanced recovery after surgery: a systematic review of qualitative studies. J Clin Nurs 26(9-10):1172-1188

7. Khan S, Wilson T, Ahmed J, et al (2010) Quality of life and patient satisfaction with enhanced recovery protocols: ERAS pathways and HQoL and patient satisfaction. Colorectal Dis 12(12):1175-1182

8. Gillis C, Gill M, Marlett N, et al (2017) Patients as partners in Enhanced Recovery After Surgery: A qualitative patient-led study. BMJ Open 7(6):e017002

9. Nelson G, Kiyang LN, Chuck A, et al (2016) Cost impact analysis of Enhanced Recovery After Surgery program implementation in Alberta colon cancer patients. Curr Oncol 23(3):221

10. Brindle M, Nelson G, Lobo DN, et al (2020) Recommendations from the ERAS® Society for standards for the development of enhanced recovery after surgery guidelines. BJS Open 4(1):157-163.

11. Ljungqvist O, Scott M, Fearon KC (2017) Enhanced Recovery After Surgery: A Review. JAMA Surg 152(3):292

12. Varadhan KK, Neal KR, Dejong CHC, et al (2010) The enhanced recovery after surgery (ERAS) pathway for patients undergoing major elective open colorectal surgery: A meta-analysis of randomized controlled trials. Clin Nutr 29(4):434-440

13. Coolsen MME, van Dam RM, van der Wilt AA, et al (2013) Systematic Review and Meta-analysis of Enhanced Recovery After Pancreatic Surgery with Particular Emphasis on Pancreaticoduodenectomies. World J Surg 37(8):1909-1918

14. Azhar RA, Bochner B, Catto J, et al (2016) Enhanced Recovery after Urological Surgery: A Contemporary Systematic Review of Outcomes, Key Elements, and Research Needs. Eur Urol 70(1):176-187

15. Yousef Y, Youssef F, Homsy M, et al (2017) Standardization of care for pediatric perforated appendicitis improves outcomes. J Pediatr Surg 52(12):1916-1920

16. Schaffzin JK, Simon K, Connelly BL, et al (2017) Standardizing preoperative preparation to reduce surgical site infections among pediatric neurosurgical patients. J Neurosurg Pediatr 19(4):399-406

17. Ryan SL, Sen A, Staggers K, et al (2014) A standardized protocol to reduce pediatric spine surgery infection: a quality improvement initiative. J Neurosurg Pediatr 14(3):259-265

18. Lyon A, Solomon MJ, Harrison JD (2014) A Qualitative Study Assessing the Barriers to Implementation of Enhanced Recovery After Surgery. World J Surg 38(6):1374-1380

19. West MA, Horwood JF, Staves S, et al (2013) Potential benefits of fast-track concepts in paediatric colorectal surgery. J Pediatr Surg 48(9):1924-1930

20. Pearson KL, Hall N (2017) What is the role of enhanced recovery after surgery in children? A scoping review. Pediatr Surg Int 33(1):43-51

21. Patel RI, Verghese ST, Hannallah RS, et al (2001) Fast-tracking children after ambulatory surgery. Patel RI, ed. Anesth Analg 92(4):918-922

22. Leeds IL, Boss EF, George JA, et al (2016) Preparing enhanced recovery after surgery for implementation in pediatric populations. J Pediatr Surg 51(12):2126-2129

23. Reismann M, Dingemann J, Wolters M, et al (2009) Fast-track concepts in routine pediatric surgery: a prospective study in 436 infants and children. Langenbecks Arch Surg 394(3):529-533

24. Short HL, Heiss KF, Burch K, et al (2017) Implementation of an enhanced recovery protocol in pediatric colorectal surgery. J Pediatr Surg 53(4):688-692

25. Segal I, Kang C, Albersheim SG et al (2014) Surgical site infections in infants admitted to the neonatal intensive care unit. J Pediatr Surg 49(3):381-384

26. Stoll BJ (2004) Neurodevelopmental and Growth Impairment Among Extremely Low-Birth-Weight Infants With Neonatal Infection. JAMA 292(19):2357

27. Matlow AG, Baker GR, Flintoft V, et al (2012) Adverse events among children in Canadian hospitals: the Canadian Paediatric Adverse Events Study. Can Med Assoc J 184(13):E709-E718

28. E Elshazzly M, Caban O, Physiology, Newborn (2019) In: StatPearls [Internet], StatPearls Publishing, Treasure Island Available from https://www.ncbi.nlm.nih.gov/books/NBK499951/

29. Mamie C, Habre W, Delhumeau C, et al (2004) Incidence and risk factors of perioperative respiratory adverse events in children undergoing elective surgery. Pediatr Anesth 14(3):218-224

30. Keenan RL (1985) Cardiac Arrest due to Anesthesia: A Study of Incidence and Causes. JAMA 253(16):2373

31. Canada NL, Mullins L, Pearo B, et al (2016) Optimizing Perioperative Nutrition in Pediatric Populations. Nutr Clin Pract 31(1):49-58

32. Gabriel MG, Wakefield CE, Vetsch J, et al (2018) The Psychosocial Experiences and Needs of Children Undergoing Surgery and Their Parents: A Systematic Review. J Pediatr Health Care 32(2):133-149

33. Kain ZN, Caldwell-Andrews AA, Mayes LC, et al (2007) Family-centered Preparation for Surgery Improves Perioperative Outcomes in Children: A Randomized Controlled Trial. Anesthesiology 106(1):65-74

34. Gibb ACN, Crosby MA, McDiarmid C, et al (2018) Creation of an Enhanced Recovery After Surgery (ERAS) Guideline for neonatal intestinal surgery patients: a knowledge synthesis and consensus generation approach and protocol study. BMJ Open 8(12):e023651

35. Guyatt GH, Oxman AD, Vist GE, et al (2008) GRADE: an emerging consensus on rating quality of evidence and strength of recommendations. BMJ 336(7650):924-926

36. Fitch K, (2001) The RAND/UCLA Appropriateness Method User’s Manual, RAND Corp, Santa Monica

37. Del Pin CA, Czyrko C, Ziegler MM, et al (1992) Management and Survival of Meconium Ileus A 30-year Review: Ann Surg 215(2):179-185

38. Jawaheer J, Khalil B, Plummer T, et al (2006) Primary resection and anastomosis for complicated meconium ileus: a safe procedure? Pediatr Surg Int 23(11):1091-1093

39. Singh M, Owen A, Gull S, et al (2006) Surgery for intestinal perforation in preterm neonates: anastomosis vs stoma. J Pediatr Surg 41(4):725-729

40. Hall NJ (2005) Resection and Primary Anastomosis Is a Valid Surgical Option for Infants With Necrotizing Enterocolitis Who Weigh Less Than 1000 g. Arch Surg 140(12):1149

41. Hillyer MM, Baxter KJ, Clifton MS, et al (2019) Primary versus secondary anastomosis in intestinal atresia. J Pediatr Surg 54(3):417-422

42. Classen DC, Evans RS, Pestotnik SL, et al (1992) The timing of prophylactic administration of antibiotics and the risk of surgical-wound infection. N Engl J Med 326(5):281-286

43. Paap CM, Nahata MC (1990) Clinical pharmacokinetics of antibacterial drugs in neonates. Clin Pharmacokinet 19(4):280-318

44. Ban KA, Minei JP, Laronga C, et al (2016) American College of Surgeons and Surgical Infection Society: Surgical Site Infection Guidelines, 2016 Update. J Am Coll Surg 224(1):59-74.

45. Bratzler DW, Dellinger EP, Olsen Km, et al (2013) Clinical practice guidelines for antimicrobial prophylaxis in surgery. Am J Health-Syst Pharm 70:195-283.

46. Meeker T (2017) Neonatal Perioperative Pathway for Newborns/Infants (≤ 12 months). Retrieved from http://www.chop.edu/clinical-pathway. https://www.chop.edu/clinical-pathway/perioperative-care-newborns-infants-clinical-pathway. Accessed August 17, 2019

47. Vu LT, Vittinghoff E, Nobuhara KK, et al (2014) Surgical site infections in neonates and infants: is antibiotic prophylaxis needed for longer than 24 h? Pediatr Surg Int 30(6):587-592

48. Walker S, Datta A, Massoumi RL, et al (2017) Antibiotic stewardship in the newborn surgical patient: A quality improvement project in the neonatal intensive care unit. Surgery 162(6):1295-1303

49. Fallat ME, Mitchell KA (1994) Random practice patterns of surgical antimicrobial prophylaxis in neonates. Pediatr Surg Int 9:479-482

50. López Sastre JB, Coto Cotallo GD, Fernández Colomer B, et al (2003) Neonatal invasive candidiasis: a prospective multicenter study of 118 cases. Am J Perinatol 20(3):153-163

51. Tander B, Baris S, Karakaya D, et al (2005) Risk factors influencing inadvertent hypothermia in infants and neonates during anesthesia. Pediatr Anesth 15(7):574-579

52. World Health Organization (1997) Thermal protection of the newborn: A practical guide. World Health Organization; Geneva: Report No.: WHO/RHT/MSM/97.2

53. Hart SR, Bordes B, Hart J, et al (2011) Unintended perioperative hypothermia. Ochsner J 11(3):259-270

54. Morehouse D, Williams L, Lloyd C, et al (2014) Perioperative Hypothermia in NICU Infants: Its Occurrence and Impact on Infant Outcomes. Adv Neonatal Care 14(3):154-164

55. Engorn BM, Kahntroff SL, Frank KM, et al (2017) Perioperative hypothermia in neonatal intensive care unit patients: effectiveness of a thermoregulation intervention and associated risk factors. Cravero J, ed. Pediatr Anesth 27(2):196-204

56. Kim P, Taghon T, Fetzer M, et al (2013) Perioperative Hypothermia in the Pediatric Population: A Quality Improvement Project. Am J Med Qual 28(5):400-406

57. Leelanukrom R, Cunliffe M (2000) Intraoperative fluid and glucose management in children. Pediatr Anesth 10(4):353-359

58. Sümpelmann R, Becke K, Brenner S, et al (2017) Perioperative intravenous fluid therapy in children: guidelines from the Association of the Scientific Medical Societies in Germany. Veyckemans F, ed. Pediatr Anesth 27(1):10-18

59. Larsson LE, Nilsson K, Niklasson A, et al (1990) Influence of fluid regimens on perioperative blood-glucose concentrations in neonates. Br J Anaesth 64(4):419-424

60. Hey E (2005) Hyperglycemia and the very preterm baby. Semin Fetal Neonatal Med 10:377-387

61. Narvey MR, Marks SD (2019) The screening and management of newborns at risk for low blood glucose. Pediatr Child Health 24(8):536-554

62. Friedman JN, Canadian Paediatric Society, Acute Care Committee (2013) Risk of acute hyponatremia in hospitalized children and youth receiving maintenance intravenous fluids. Paediatr Child Health 18(2):102-107

63. Duke T, Molyneux EM (2003) Intravenous fluids for seriously ill children: time to reconsider. The Lancet 362(9392):1320-1323

64. Ceelie I, de Wildt SN, van Dijk M, et al (2013) Effect of Intravenous Paracetamol on Postoperative Morphine Requirements in Neonates and Infants Undergoing Major Noncardiac Surgery: A Randomized Controlled Trial. JAMA 309(2):149

65. Allegaert K, Palmer GM, Anderson BJ (2011) The pharmacokinetics of intravenous paracetamol in neonates: size matters most. Arch Dis Child 96(6):575-580

66. Allegaert K, Rayyan M, De Rijdt T, et al (2008) Hepatic tolerance of repeated intravenous paracetamol administration in neonates. Pediatr Anesth 18(5):388-392

67. Palmer GM, Atkins M, Anderson BJ, et al (2008) I.V. acetaminophen pharmacokinetics in neonates after multiple doses. Br J Anaesth 101(4):523-530

68. van Dijk M, Bouwmeester NJ, Duivenvoorden HJ, et al (2002) Efficacy of continuous versus intermittent morphine administration after major surgery in 0–3-year-old infants; a double-blind randomized controlled trial: Pain 98(3):305-313

69. Bouwmeester NJ, Hop WCJ, van Dijk M, et al (2003) Postoperative pain in the neonate: age-related differences in morphine requirements and metabolism. Intensive Care Med 29(11):2009-2015

70. Howard RF, Lloyd-Thomas A, Thomas M, et al (2010) Nurse-controlled analgesia (NCA) following major surgery in 10 000 patients in a children’s hospital. Pediatr Anesth 20(2):126-134

71. Bouwmeester NJ, Anand KJS, van Dijk M, et al (2001) Hormonal and metabolic stress responses after major surgery in children aged 0–3 years: a double-blind, randomized trial comparing the effects of continuous versus intermittent morphine. Br J Anaesth 87(3):390-399

72. Saarenmaa E, Huttunen P, Leppäluoto J, et al Advantages of fentanyl over morphine in analgesia for ventilated newborn infants after birth: A randomized trial. J Pediatr 134(2):144-150

73. Somri M, Tome R, Yanovski B, et al (2007) Combined spinal-epidural anesthesia in major abdominal surgery in high-risk neonates and infants. Pediatr Anesth 17(11):1059-1065

74. Somri M, Coran AG, Mattar I, et al (2011) The postoperative occurrence of cardio-respiratory adverse events in small infants undergoing gastrointestinal surgery: a prospective comparison of general anesthesia and combined spinal-epidural anesthesia. Pediatr Surg Int 27(11):1173-1178

75. Somri M, Matter I, Parisinos CA, et al (2012) The effect of combined spinal-epidural anesthesia versus general anesthesia on the recovery time of intestinal function in young infants undergoing intestinal surgery: a randomized, prospective, controlled trial. J Clin Anesth 24(6):439-445

76. Fredrickson MJ, Seal P (2009) Ultrasound-guided Transversus Abdominis Plane Block for Neonatal Abdominal Surgery. Anaesth Intensive Care 37(3):469-472

77. Jacobs A, Bergmans E, Arul GS, et al (2011) The transversus abdominis plane (TAP) block in neonates and infants - results of an audit: Correspondence. Pediatr Anesth 21(10):1078-1080

78. Carbajal R (2008) Epidemiology and Treatment of Painful Procedures in Neonates in Intensive Care Units. JAMA 300(1):60

79. Grunau RE, Oberlander TF, Whitfield MF, et al (2001) Demographic and Therapeutic Determinants of Pain Reactivity in Very Low Birth Weight Neonates at 32 Weeks’ Postconceptional Age. Pediatrics 107(1):105-112

80. Pineda R, Guth R, Herring A, et al (2017) Enhancing sensory experiences for very preterm infants in the NICU: an integrative review. J Perinatol 37(4):323-332

81. Walter-Nicolet E, Annequin D, Biran V, et al (2010) Pain Management in Newborns: From Prevention to Treatment. Pediatr Drugs 12(6):353-365

82. Stevens B, Yamada J, Ohlsson A, et al (2016) Sucrose for analgesia in newborn infants undergoing painful procedures. Cochrane Neonatal Group, ed. Cochrane Database Syst Rev 7:CD001069

83. McCullough S, Halton T, Mowbray D, et al (2007) Lingual sucrose reduces the pain response to nasogastric tube insertion: a randomised clinical trial. Arch Dis Child - Fetal Neonatal Ed 93(2):F100-F103

84. Ravishankar A, Thawani R, Dewan P, et al (2014) Oral dextrose for analgesia in neonates during nasogastric tube insertion: A randomised controlled trial: Oral dextrose for analgesia in neonates. J Paediatr Child Health 50(2):141-145

85. Goobie S, Faraoni D, Zurakowski D (2016) Association of preoperative anemia with postoperative mortality in neonates. JAMA Pediatr 170(9):855-862

86. New HV, Stanworth S, Engelfriet C, et al (2009) Neonatal transfusions. Vox Sang 96(1):62

87. Lau W, Neonatal and Pediatric Transfusion (2017) In: Clarke G, Charge S (eds) Canadian Blood Services’ Clinical Guide to Transfusion. Retrieved from <https://professionaleducation.blood.ca/en/transfusion/guide-clinique/neonatal-and-pediatric-transfusion>

88. Whyte RK, Jeffries AL, Canadian Pediatric Society, Fetus and Newborn Committee (2014) Red blood cell transfusion in newborn infants. Pediatr Child Health 19(4):213-217

89. Mancuso MP, Dziadkowiec O, Kleiner C, et al (2016) Crew Resource Management for Obstetric and Neonatal Teams to Improve Communication During Cesarean Births. J Obstet Gynecol Neonatal Nurs 45(4):502-514

90. Brodsky D, Gupta M, Quinn M, et al (2013) Building collaborative teams in neonatal intensive care. BMJ Qual Saf 22(5):374-382

91. Catchpole KR, De Leval MR, Mcewan A, et al (2007) Patient handover from surgery to intensive care: using Formula 1 pit-stop and aviation models to improve safety and quality. Pediatr Anesth 17(5):470-478

92. Zavalkoff SR, Razack SI, Lavoie J, et al (2011) Handover after pediatric heart surgery: A simple tool improves information exchange. Pediatr Crit Care Med 12(3):309-313

93. Lagoo J, Lopushinsky SR, Haynes AB, et al (2017) Effectiveness and meaningful use of paediatric surgical safety checklists and their implementation strategies: a systematic review with narrative synthesis. BMJ Open 7(10):e016298

94. Segall N, Bonifacio AS, Schroeder RA, et al (2012) Can We Make Postoperative Patient Handovers Safer? A Systematic Review of the Literature: Anesth Analg 115(1):102-115

95. Singer SJ, Molina G, Li Z, et al (2016) Relationship Between Operating Room Teamwork, Contextual Factors, and Safety Checklist Performance. J Am Coll Surg 223(4):568-580.e2

96. Franck LS, Oulton K, Nderitu S, et al (2011) Parent Involvement in Pain Management for NICU Infants: A Randomized Controlled Trial. Pediatrics 128(3):510-518

97. Browne JV, Talmi A (2005) Family-Based Intervention to Enhance Infant–Parent Relationships in the Neonatal Intensive Care Unit. J Pediatr Psychol 30(8):667-677

98. Glazebrook C, Marlow N, Israel C, et al (2007) Randomised trial of a parenting intervention during neonatal intensive care. Arch Dis Child - Fetal Neonatal Ed 92(6):F438-F443

90. Voos KC, Ross G, Ward MJ, et al (2011) Effects of implementing family-centered rounds (FCRs) in a neonatal intensive care unit (NICU). J Matern Fetal Neonatal Med 24(11):1403-1406

100. Weis J, Zoffmann V, Egerod I. Enhancing person-centred communication in NICU: a comparative thematic analysis: Communication in NICU. *Nurs Crit Care*. 2015;20(6):287-298. doi:10.1111/nicc.12062

101. Abdel-Latif ME, Boswell D, Broom M, Smith J, Davis D. Parental presence on neonatal intensive care unit clinical bedside rounds: randomised trial and focus group discussion. *Arch Dis Child - Fetal Neonatal Ed*. 2015;100(3):F203-F209. doi:10.1136/archdischild-2014-306724

102. Weiss S, Goldlust E, Vaucher YE (2010) Improving parent satisfaction: an intervention to increase neonatal parent–provider communication. J Perinatol 30(6):425-430

103. Globus O, Leibovitch L, Maayan-Metzger A, et al (2016) The use of short message services (SMS) to provide medical updating to parents in the NICU. J Perinatol 36(9):739-743

104. Kerr S, King C, Hogg R, et al (2017) Transition to parenthood in the neonatal care unit: a qualitative study and conceptual model designed to illuminate parent and professional views of the impact of webcam technology. BMC Pediatr 17(1):158

105. Provenzi L, Santoro E (2015) The lived experience of fathers of preterm infants in the Neonatal Intensive Care Unit: a systematic review of qualitative studies. J Clin Nurs 24(13-14):1784-1794

106. Penticuff JH, Arheart KL (2005) Effectiveness of an Intervention to Improve Parent-Professional Collaboration in Neonatal Intensive Care: J Perinat Neonatal Nurs 19(2):187-202

107. Sisson H, Jones C, Williams R, et al (2015) Metaethnographic Synthesis of Fathers’ Experiences of the Neonatal Intensive Care Unit Environment During Hospitalization of Their Premature Infants. J Obstet Gynecol Neonatal Nurs 44(4):471-480

108. Sneath N (2009) Discharge Teaching in the NICU: Are Parents Prepared? An Integrative Review of Parents’ Perceptions. Neonatal Netw 28(4):237-246

109. Franck LS, McNulty A, Alderdice F (2017) The Perinatal-Neonatal Care Journey for Parents of Preterm Infants: What Is Working and What Can Be Improved. J Perinat Neonatal Nurs 31(3):244-255

110. Schweitzer M, Aucoin J, Docherty SL, et al (2014) Evaluation of a Discharge Education Protocol for Pediatric Patients With Gastrostomy Tubes. J Pediatr Health Care 28(5):420-428

111. Pfander S, Bradley-Johnson S (1990) Effects of an Intervention Program and its Components on NICU Infants. Child Health Care 19(3):140-146

112. Ingram JC, Powell JE, Blair PS, et al (2016) Does family-centred neonatal discharge planning reduce healthcare usage? A before and after study in South West England. BMJ Open 6(3):e010752

113. Raines DA (2017) Simulation as Part of Discharge Teaching for Parents of Infants in the Neonatal Intensive Care Unit: MCN Am J Matern Nurs 42(2):95-100

114. Ekingen G, Ceran C, Guvenc BH, et al (2005) Early enteral feeding in newborn surgical patients. Nutrition 21(2):142-146

115. Prasad GR, Subba Rao JV, Aziz A, et al (2018) Early Enteral Nutrition in Neonates Following Abdominal Surgery. J Neonatal Surg 7(2):21

116. Dunn L, Hulman S, Weiner J, et al (1988) Beneficial effects of early hypocaloric enteral feeding on neonatal gastrointesting function: Preliminary report of a randomized trial. J Pediatr 112(4):622-629

117. Terrin G, Passariello A, Canani RB, et al (2009) Minimal enteral feeding reduces the risk of sepsis in feed-intolerant very low birth weight newborns. Acta Paediatr 98(1):31-35

118. Morgan J, Young L, McGuire W (2014) Delayed introduction of progressive enteral feeds to prevent necrotising enterocolitis in very low birth weight infants. Cochrane Neonatal Group, ed. Cochrane Database Syst Rev 12:CD001970

119. Varma S, Bartlett EL, Nam L, et al (2019) Use of Breast Milk and Other Feeding Practices Following Gastrointestinal Surgery in Infants. J Pediatr Gastroenterol Nutr 68(2):264-271

120. Agostoni C, Buonocore G, Carnielli V, et al (2010) Enteral Nutrient Supply for Preterm Infants: Commentary From the European Society of Paediatric Gastroenterology, Hepatology and Nutrition Committee on Nutrition. J Pediatr Gastroenterol Nutr 50(1):85-91

121. Meredith-Dennis L, Xu G, Goonatilleke E, et al (2018) Composition and Variation of Macronutrients, Immune Proteins, and Human Milk Oligosaccharides in Human Milk From Nonprofit and Commercial Milk Banks. J Hum Lact 34(1):120-129

122. Quigley M, Embleton ND, McGuire W (2018) Formula versus donor breast milk for feeding preterm or low birth weight infants. Cochrane Neonatal Group, ed. Cochrane Database Syst Rev 6:CD002971

123. Lambert DK, Christensen RD, Henry E, et al (2007) Necrotizing enterocolitis in term neonates: data from a multihospital health-care system. J Perinatol 27(7):437-443

124. Kleessen B, Bunke H, Tovar K, et al (1995) Influence of two infant formulas and human milk on the development of the faecal flora in newborn infants. Acta Paediatr 84(12):1347-1356

125. Butterworth SA, Lalari V, Dheensaw K (2014) Evaluation of sodium deficit in infants undergoing intestinal surgery. J Pediatr Surg 49(5):736-740

126. Mansour F, Petersen D, De Coppi P, et al (2014) Effect of sodium deficiency on growth of surgical infants: a retrospective observational study. Pediatr Surg Int 30(12):1279-1284

1127. Lau ECT, Fung ACH, Wong KKY, et al (2016) Beneficial effects of mucous fistula refeeding in necrotizing enterocolitis neonates with enterostomies. J Pediatr Surg 51(12):1914-1916

128. Wong KKY, Lan LCL, Lin SCL, et al (2004) Mucous Fistula Refeeding in Premature Neonates With Enterostomies. J Pediatr Gastroenterol Nutr 39(1):43-45

129. Koike Y, Uchida K, Nagano Y, et al (2016) Enteral refeeding is useful for promoting growth in neonates with enterostomy before stoma closure. J Pediatr Surg 51(3):390-394

130. Al-Harbi K, Walton JM, Gardner V, et al (1999) Mucous fistula refeeding in neonates with short bowel syndrome. J Pediatr Surg 34(7):1100-1103

131. Gause CD, Hayashi M, Haney C, et al (2016) Mucous fistula refeeding decreases parenteral nutrition exposure in postsurgical premature neonates. J Pediatr Surg 51(11):1759-1765

132. Haddock CA, Stanger JD, Albersheim SG, et al (2015) Mucous fistula refeeding in neonates with enterostomies. J Pediatr Surg 50(5):779-782

133. Richardson L, Banerjee S, Rabe H (2006) What Is the Evidence on the Practice of Mucous Fistula Refeeding in Neonates With Short Bowel Syndrome? J Pediatr Gastroenterol Nutr 43(2):267-270
